# PRMT1-catalyzed NUSAP1 methylation enhances Notch2 signaling and 5-FU resistance in gastric cancer

**DOI:** 10.1038/s41419-025-07723-9

**Published:** 2025-05-20

**Authors:** Suting Jiang, Guoli Li, Shihan Peng, Shitong Chen, Yi Pang, Hongjuan Cui, Feng Wang

**Affiliations:** 1 Chongqing Key Laboratory of Development and Utilization of Genuine Medicinal Materials in Three Gorges Reservoir Area, Chongqing Three Gorges Medical College, Chongqing, China; 2 Chongqing Engineering Research Center of Antitumor Natural Drugs, Chongqing Three Gorges Medical College, Chongqing, China; 3 State Key Laboratory of Resource Insects, Medical Research Institute, Southwest University, Chongqing, China; 4 Jinfeng Laboratory, Chongqing, China

**Keywords:** Gastric cancer, Oncogenes, Predictive markers, Oncogenes

## Abstract

5-Fluorouracil (5-FU) resistance remains a significant challenge in the treatment of gastric cancer, limiting its clinical efficacy. Our study identifies NUSAP1, a nucleolar and spindle-associated protein, as a key driver of 5-FU resistance in gastric cancer. Proteomic analyses of 5-FU-resistant gastric cancer cell lines revealed that NUSAP1 is significantly upregulated, and functional studies demonstrated its essential role in promoting resistance, proliferation, migration, invasion, and tumor growth. Mechanistic investigations revealed that NUSAP1 undergoes asymmetric dimethylation (ADMA) at R418 and R422, mediated by PRMT1, with the R422 site being critical for its function. NUSAP1 interacts with the PEST domain of Notch2 through its R422 site, inhibiting Notch2 ubiquitination and stabilizing its expression, thereby activating the Notch2 signaling pathway. This pathway is closely linked to gastric cancer progression and chemoresistance. Inhibition of PRMT1 or mutation of the R422 site abrogated NUSAP1’s ability to stabilize Notch2 and regulate downstream signaling. These findings unveil a novel mechanism by which NUSAP1 promotes 5-FU resistance in gastric cancer and highlight the therapeutic potential of targeting the NUSAP1-Notch2 axis or PRMT1 in overcoming chemoresistance.

## Introduction

Despite significant progress in tumor drug therapy, drug resistance remains a major obstacle to effective treatment [1]. The extensive plasticity of gene expression and epigenetic alterations in cancer cells contribute to the development of resistance [2]. Therefore, identifying genes differentially expressed in drug-resistant cells and understanding the proteins and epigenetic modifications supporting resistance are critical steps toward developing effective strategies for preventing and targeting resistance.

5-Fluorouracil (5-FU) is a primary chemotherapeutic agent used to treat various solid malignancies, including colorectal, gastric, pancreatic, bladder, and breast cancers [3–5]. 5-FU exerts its cytotoxic effects primarily by inhibiting thymidylate synthase (TS), disrupting essential biosynthetic processes, or incorporating its metabolites into RNA and DNA [6]. Clinically, 5-FU is often combined with leucovorin and capecitabine to enhance therapeutic efficacy [7]. However, since 5-FU primarily targets highly proliferative cancer cells, quiescent and poorly differentiated cancer stem cells (CSCs) within the tumor microenvironment (TME) can evade its effects and survive. Studies suggest that these CSCs significantly contribute to 5-FU resistance [8, 9]. Furthermore, tumor cells resistant to 5-FU exhibit upregulated expression of stem cell markers (e.g., Notch, CD44, ALDHA1, Oct4, and Sox2) and demonstrate enhanced migratory, invasive, and colony-forming capabilities [10], further supporting this conclusion. Elucidating the underlying mechanisms of 5-FU resistance is therefore critical for improving clinical treatment.

NUSAP1 (nucleolar and spindle-associated protein 1) is a nucleolar-spindle-associated protein involved in spindle microtubule organization [11]. Subsequent studies have revealed that NUSAP1 is associated with tumor cell proliferation and metastasis. Silencing NUSAP1 expression inhibits the proliferation, migration, and invasion of colorectal and glioblastoma cells by downregulating DNMT1 or TOP2A [12, 13]. Additionally, NUSAP1 has been shown to regulate the BTG2/PI3K/Akt signaling pathway [14]. Research has demonstrated that NUSAP1 stabilizes ATR protein expression, promoting glioblastoma proliferation and resistance to temozolomide [15]. Furthermore, NUSAP1 suppresses ubiquitin-mediated degradation of YAP1 and ANXA2, driving gastric cancer progression [16, 17]. Lactate-driven nuclear localization of NUSAP1 is critical for its function, as nuclear NUSAP1 recruits the JUNB-FRA1-FRA2 transcriptional complex to the DESMIN promoter, activating DESMIN transcription and promoting cancer-associated fibroblast (CAF) activation [18]. NUSAP1 also forms a transcriptional regulatory complex with C-Myc and HIF-1α at the LDHA promoter, enhancing its expression [19]. These findings underscore NUSAP1’s dual role in gene transcription regulation and protein stability, highlighting its robust biological functions.

In this study, we identified NUSAP1 upregulation in 5-FU-resistant cells using proteomics. Inhibition of NUSAP1 expression enhanced tumor cell sensitivity to 5-FU. We further identified multiple arginine methylation sites on NUSAP1 and demonstrated that PRMT1-mediated R422me2 modification facilitates its interaction with Notch2, stabilizing Notch2 protein expression and promoting 5-FU resistance in gastric cancer cells. Our findings provide preliminary evidence supporting NUSAP1 and its R422 methylation as potential biomarkers for predicting poor prognosis and resistance to 5-FU therapy in gastric cancer patients.

## Materials and methods

### Cell culture

Gastric cancer (GC) cell lines (BGC-823 and SGC-7901) and human embryonic renal cell line HEK293FT were obtained from American Type Culture Collection (ATCC, Beijing, China), 5-FU-resistant GC cell lines (BGC-823-5-FU-R and SGC-7901-5-FU-R) were obtained from Meixuan Biotechnology (Shanghai, China). GC cell lines and 5-FU-resistant GC cell lines were cultured in Park Memorial Institute-1660 (RPMI-1640, Gibco) supplementer with 10% fetal bovine serum (FBS) and 1% penicillin and streptomycin (P/S). HEK293FT cells were maintained in Dulbecco’s Modified Eagle’s Medium (DMEM, Gibco) supplemented with 10% FBS, 1% P/S, 4 mM L-glutamine, 1 mM sodium pyruvate, 0.1 mM nonessential amino acids (MEM), and 0.5 mg/mL G418. All cell lines were cultured with 5% CO2 at 37°C in a humidified incubator.

### Transfection and infection

Human NUSAP1 shRNA (short hairpin RNA) and PRMT1 shRNA were purchased from BGI. The shNUSAP1 and shPRMT1 sequences (Table 1) were inserted into a pLKO.1 vector. The constructed plasmid was combined with 0.6 μg of pLP1, pLP2 and pLP/VSVG plasmids and transfected into HEK293FT cells using Lipofectamine 2000 transfection agent. After culturing for 48 h, the supernatant was collected.

**Table 1:** List of shRNA primers used in this study.

Name	Sequence (5’–3’)	
shNUSAP1-1#	F:CCGGGAACTGAAGCAGCCCATCAATCTCGAGATTGATGGGCTGCTTCAGTTCTTTTTG	R:AATTCAAAAAGAACTGAAGCAGCCCATCAATCTCGAGATTGATGGGCTGCTTCAGTTC
shNUSAP1-2#	F:CCGGGAGGGCAACCAAGTTGTTAAACTCGAGTTTAACAACTTGGTTGCCCTCTTTTTG	R:AATTCAAAAAGAGGGCAACCAAGTTGTTAAACTCGAGTTTAACAACTTGGTTGCCCTC
shPRMT1-1#	F:CCGGGTGTTCCAGTATCTCTGATTACTCGAGTAATCAGAGATACTGGAACACTTTTTG	R:AATTCAAAAAGTGTTCCAGTATCTCTGATTACTCGAGTAATCAGAGATACTGGAACAC
shPRMT1-2#	F:CCGGTGAGCGTTCCTAGGCGGTTTCCTCGAGGAAACCGCCTAGGAACGCTCATTTTTG	R:AATTCAAAAATGAGCGTTCCTAGGCGGTTTCCTCGAGGAAACCGCCTAGGAACGCTCA

This table lists the shRNA primer sequences used to silence NUSAP1 and PRMT1 in the study.

The virus-containing supernatant was supplemented with 4 μg/mL polybrene and used for infection by incubation for 24 h. After two rounds of infection, the cells were cultured with 2 mg/mL puromycin for stable cell line selection. After two days of selection, the stable cell lines were harvested for subsequent experiments.

### Western blotting and antibodies

The collected cell pellets were lysed using RIPA lysis buffer containing 1% PMSF and centrifuged at 12,000 × *g* for 10 min at 4 °C to remove the precipitate. The protein concentration was then determined using a BSA protein assay kit (Beyotime, P0009), and the lysates were heat-inactivated. Proteins were separated by SDS-PAGE and subsequently transferred onto PVDF membranes. The membranes were blocked with 5% skim milk at room temperature for 2 h and incubated overnight at 4 °C with primary antibodies as listed in Table 2. On the following day, the primary antibodies were recovered, and the PVDF membranes were washed three times with TBST buffer. The membranes were then incubated with homologous secondary antibodies for 2 h at room temperature. Protein signals were detected using ECL reagent (Clinx Science) and visualized with a western blot detection system (Clinx Science). Each western blot experiment was performed independently at least three times to ensure reproducibility.

**Table 2:** List of antibodies.

List of Antibodies		
Antibody	Company	Product Code
PRMT5	Abcam	Ab109451
PRMT1	Abcam	Ab190892
GST-Tag	Abcam	Ab252882
His-Tag	Abcam	Ab5000
Tubulin	Abcam	Ab6160
ADMA	Cell Signaling Technology	13522
SDMA	Cell Signaling Technology	13222
Notch2	Cell Signaling Technology	4530
C-Myc	Cell Signaling Technology	18583
CyclinD3	Cell Signaling Technology	2936
CDKN1A	Cell Signaling Technology	2947
HES1	Cell Signaling Technology	11988
Myc-Tag(Mouse)	Cell Signaling Technology	2276
Myc-Tag(Rabbit)	Cell Signaling Technology	3946
Flag-Tag(Mouse)	Cell Signaling Technology	8146
Flag-Tag(Rabbit)	Cell Signaling Technology	14793
HA-Tag	Cell Signaling Technology	3724
HES5	Proteintech	22666-1-AP
HEY1	Proteintech	19929-1-AP
NUSAP1	Proteintech	12024-1-AP
Tubulin	Proteintech	66031-1-lg

This table summarizes all antibodies used for western blotting and immunofluorescence.

### Cell viability assay

MTT(3-(4, 5-dimethylthiazol-2)-2, 5-diphenyltetrazolium bromide salt) was used in Cell viability assay. 1 × 10^3^ cells per well in a 96-well plate and culturing for 24 h to ensure cell adhesion. Subsequently, 20 μL of MTT solution was added to each well, and the plate was incubated at 37 °C for 2 h. After incubation, the culture medium was removed, and 100 μL of DMSO was added to each well. The plate was shaken for 10 min, and the absorbance at 560 nm was measured using a microplate reader. Absorbance was measured every other day for a total of four measurements, and the absorbance values were plotted as a line graph.

### EDU assay

Cells were incubated with 10 μM EDU (5-Ethynyl-2’-deoxyuridine) working solution at 37 °C for 2 h. After incubation, the EDU working solution was removed, and the cells were washed with PBS. The cells were then fixed with 4% paraformaldehyde at room temperature for 15 min. Following fixation, the paraformaldehyde solution was removed, and the cells were washed with PBS. Subsequently, PBS containing 0.3% Triton X-100 was added, and the cells were incubated at room temperature for 15 min to permeabilize the membrane. After removing the permeabilization solution and washing with PBS, 100 μL of Click Additive Solution (Beyotime, COO78L) was added to each well, and the cells were incubated in the dark at room temperature for 30 min. The Click Additive Solution was then removed, and the cells were washed with PBS. DAPI was added, and the cells were incubated in the dark at room temperature for 5 min. After removing the DAPI staining solution and washing with PBS, fluorescence microscopy was used to observe and capture images.

### Migration and Invasion assays

For the migration assay, 8 × 10^4^ cells in 200 μL of FBS-free medium were plated in a 24-well plate cell culture insert, and RPMI-1640 medium containing 10% FBS was added to the bottom of the insert. After incubation for 36 h, the cells were fixed in 4% paraformaldehyde for 20 min and then stained with crystal violet blue for 20 min. After washing three times with PBS, the cells on the upper surface of the insert were removed with a cotton swab. Then, the polyester (PET) insert membrane was exfoliated and examined with a microscope. For the invasion assay, 50 μL of Matrigel was added to each insert before the cells were plated. Then, the insert was incubated at 37 °C in a humidified incubator until the Matrigel had solidified. Then, the invasion assay was performed following the same protocol followed for the migration assay.

### Soft agar assay and subcutaneous xenograft

The lower layer was formed by growth medium and 0.6% agarose to perform vitro soft agar assay. 1 × 10^3^ cells were cultured in upper layer consisting of growth medium with 0.3% agarose. After cultured for 3-4 weeks, colonies were photographed and recorded.

Briefly, for in vivo subcutaneous xenograft assay, each 4-week-old NOD/SCID female mice was injected with 4 × 10^6^ cells. Randomization and single-blinding were used for measurement. All the mice were raised under specific pathogen-free (SPF) conditions. After one month of growth, the mice were euthanized, and tumors were removed and weighted. Animal experiments were approved by the IACUC of Three Gorges Medical College and carried out in conformity to the Guide for the Care and Use of Laboratory Animals (Ministry of Science and Technology of China, 2006).

### Immunoprecipitation (IP) and mass spectrometry (MS)

Cells were lysed by immunoprecipitation lysis buffer containing protease inhibitor cocktail for 1 h. Then centrifuged (12,000 × *g* 15 min 4 °C) and separated the supernatant. Supernatant was incubated with anti MYC Nanobody Magarose Beads (AlpaLifeBio, KTSM1365) at 4 °C overnight. After the incubation, anti MYC Nanobody Magarose Beads was washed for five times with ice-cold PBS buffer. After boiling the anti MYC Nanobody Magarose Beads for 10 min, bound proteins were recovered in sample loading buffer, and used to western boltting assay. The NUSAP1-binding proteins were identified by liquid chromatography-tandem mass spectrometry (Bioprofile, Shanghai, China).

### Proximity ligation assay (PLA)

After transient transfection, 3 × 10^4^ cells were grown on coverslips in 24-well plates in preparation for a PLA. The cells were fixed in 4% paraformaldehyde for 20 min and permeabilized in 0.3% Triton X-100 for 15 min at room temperature. Then, after blocking in 5% goat serum for 1 h at room temperature, the coverslips were incubated with primary antibodies overnight at 4 °C. After washing with wash buffer A three times, the coverslips were incubated with PLA probes (DUO92001 and DUO92005, Sigma) for 1 h at 37 °C. Then, the coverslips were incubated with ligation-ligase solution (DUO92008, Sigma) for 30 min at 37 °C and amplification-polymer solution (DUO92008, Sigma) for 100 min at 37 °C. Finally, the signal was captured by confocal microscopy (FluoView FV1000, Olympus, Japan) after nuclear and tubulin staining.

### GST pull down

Briefly, GST-tagged fusion protein (GST-NUSAP1-WT) and His-tagged fusion proteins (His-PRMT1,His-PRMT5 and His-N2ICD) were expressed in *Escherichia* *coli* with 0.5 mM IPTG induction at 16 °C for 12–16 h. Bacterial lysates were prepared in lysis buffer (20 mM Tris-HCl, pH 7.5, 150 mM NaCl, 1 mM EDTA, 1% Triton X-100) and incubated with glutathione-Sepharose beads at 4 °C for 2 h. The GST-bound beads were washed with binding buffer (20 mM Tris-HCl, pH 7.5, 150 mM NaCl, 0.5% Triton X-100) and incubated with purified prey proteins or cell lysates containing the prey protein at 4 °C for 4 h. After binding, beads were washed 5 times with washing buffer (20 mM Tris-HCl, pH 7.5, 300 mM NaCl, 0.5% Triton X-100). Bound proteins were eluted by boiling in SDS-PAGE sample buffer and analyzed by SDS-PAGE followed by Western blotting using specific antibodies. Beads incubated with GST alone were used as a negative control to assess nonspecific binding.

### Statistical analysis

All experiments were carried out in triplicates and statistical parameters including the sample size and the significance analysis are specified in figure legends. Data from at least three experiments were detected as mean values ± standard deviation. Two-tailed Student’s *t*-test was performed for paired samples, following normal distribution with different but similar SDs. All analysis was performed in GraphPad Prism 5. The *P* value < 0.05 was considered significant.

## Results

### NUSAP1 promotes 5-FU resistance in gastric cancer

**Fig. 1:**
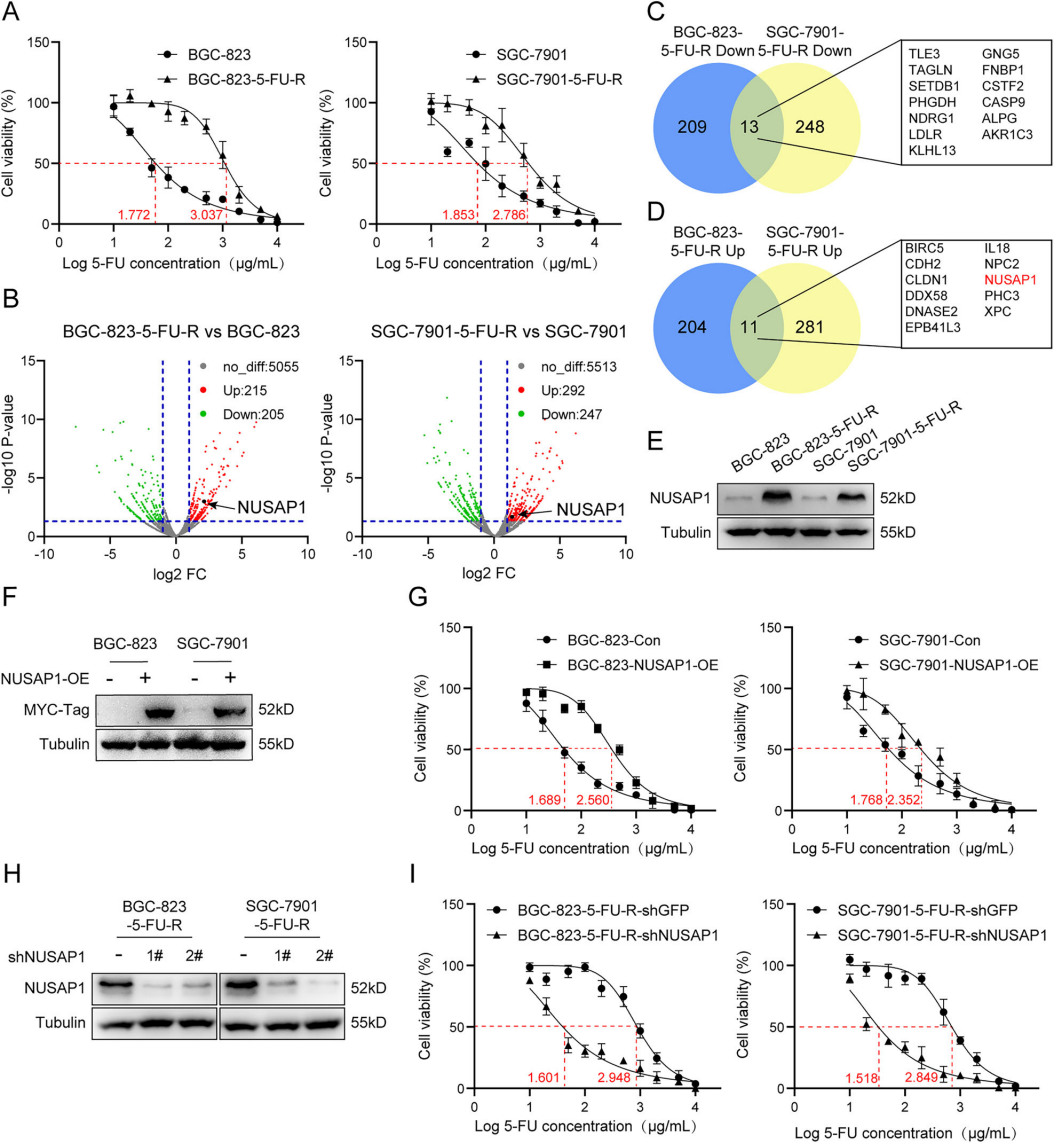
NUSAP1 promotes 5-FU resistance in gastric cancer. **A** IC50 assays were performed to evaluate the sensitivity of BGC-823 and SGC-7901 parental and 5-FU-resistant (5-FU-R) cell lines to 5-FU. **B** Volcano plot analysis showing differentially expressed genes (−log10 P-value < 15, log2 −10 < FC < -1 or 1 < FC < 10) between 5-FU-resistant and parental gastric cancer cells. Upregulated and downregulated genes are highlighted. Venn diagrams showing the overlap of significantly downregulated (**C**) and upregulated (**D**) genes in BGC-823-5-FU-R and SGC-7901-5-FU-R cell lines compared to their parental counterparts. **E** Western blot analysis of NUSAP1 protein expression in BGC-823, SGC-7901, and their 5-FU-resistant derivatives. Tubulin served as a loading control. **F** Western blot analysis confirming overexpression of NUSAP1 in BGC-823 and SGC-7901 cells transfected with a NUSAP1 overexpression plasmid (NUSAP1-OE). MYC-tag was used to validate NUSAP1 expression.**G** IC50 assays were performed to determine the effect of NUSAP1 overexpression on 5-FU sensitivity in BGC-823 and SGC-7901 cells. **H** Western blot analysis of NUSAP1 protein levels in BGC-823-5-FU-R and SGC-7901-5-FU-R cells transfected with shNUSAP1-1#, shNUSAP1-2#, or shGFP control (negative control). **I** IC50 assays were performed to evaluate the effect of NUSAP1 knockdown on 5-FU sensitivity in BGC-823-5-FU-R and SGC-7901-5-FU-R cells.

To investigate the mechanism of 5-FU resistance in gastric cancer (GC) cells, the human GC cell lines BGC-823 and SGC-7901 were induced to develop resistance to 5-FU using a low-concentration gradient combined with high-dose intermittent shock. This method resulted in the establishment of 5-FU-resistant GC cell lines (BGC-823-5-FU-R and SGC-7901-5-FU-R), obtained from Shanghai MEIXUAN [20]. The 24 h IC50 assay results demonstrated significantly increased resistance to 5-FU in the two resistant cell lines (Fig. 1A).

To explore the changes in gene expression in 5-FU-resistant cells, we performed proteomic analysis. In BGC-823-5-FU-R, 215 upregulated and 205 downregulated proteins were identified, while in SGC-7901-5-FU-R, 292 upregulated and 247 downregulated proteins were detected (Fig. 1B). Intersection analysis of these differentially expressed proteins revealed 13 proteins commonly downregulated (Fig. 1C) and 11 proteins commonly upregulated in both resistant cell lines (Fig. 1D).

Using the GEPIA2 database (http://gepia2.cancer-pku.cn/#index), we analyzed the expression of these 11 upregulated proteins across multiple tumor types. BIRC5, CLDN1, DDX58, IL18, NPC2, NUSAP1, and PHC3 were found to have higher expression in gastric cancer tissues compared to normal tissues, and their expression was also elevated in various other cancer types. In contrast, CDH2 was downregulated in gastric cancer, while EPB41L3, DNASE2, and XPC showed no significant expression differences between gastric cancer and normal tissues (Fig. S1). Further analysis using the TCGA database revealed that BIRC5, CLDN1, DNASE2, IL18, NUSAP1, XPC, NPC2, and DDX58 were upregulated in gastric cancer and closely associated with tumor malignancy (Fig. S2). Among these, NUSAP1 and NPC2 showed the most significant differences. Comparative analysis showed that NPC2 was upregulated in 11 tumor types, while NUSAP1 was upregulated in as many as 22 tumor types (Fig. S2A). Additionally, data from the GEO database (GSE171054) indicated that NUSAP1 expression was upregulated in 5-FU-resistant gastric cancer patients and that its high expression was associated with poor prognosis in these patients (Fig. S2B). Therefore, we further examined the expression of NUSAP1 in 5-FU-resistant cells and confirmed a significant increase in its expression in 5-FU-resistant GC cells (Fig. 1E).

To explore whether NUSAP1 influences sensitivity to 5-FU, we overexpressed MYC-tagged NUSAP1 protein in normal GC cells (Fig. 1F). IC50 assays showed that NUSAP1 overexpression significantly decreased sensitivity to 5-FU (Fig. 1G). Conversely, when NUSAP1 expression was silenced in 5-FU-resistant GC cells using shRNA vectors (Fig. 1H), the cells’ resistance to 5-FU was significantly reduced (Fig. 1I).

In summary, we found that NUSAP1 is not only upregulated in 5-FU-resistant gastric cancer cells but also enhances cellular resistance to 5-FU.

### NUSAP1 promotes proliferation and tumor growth in 5-FU-resistant gastric cancer cells

**Fig. 2:**
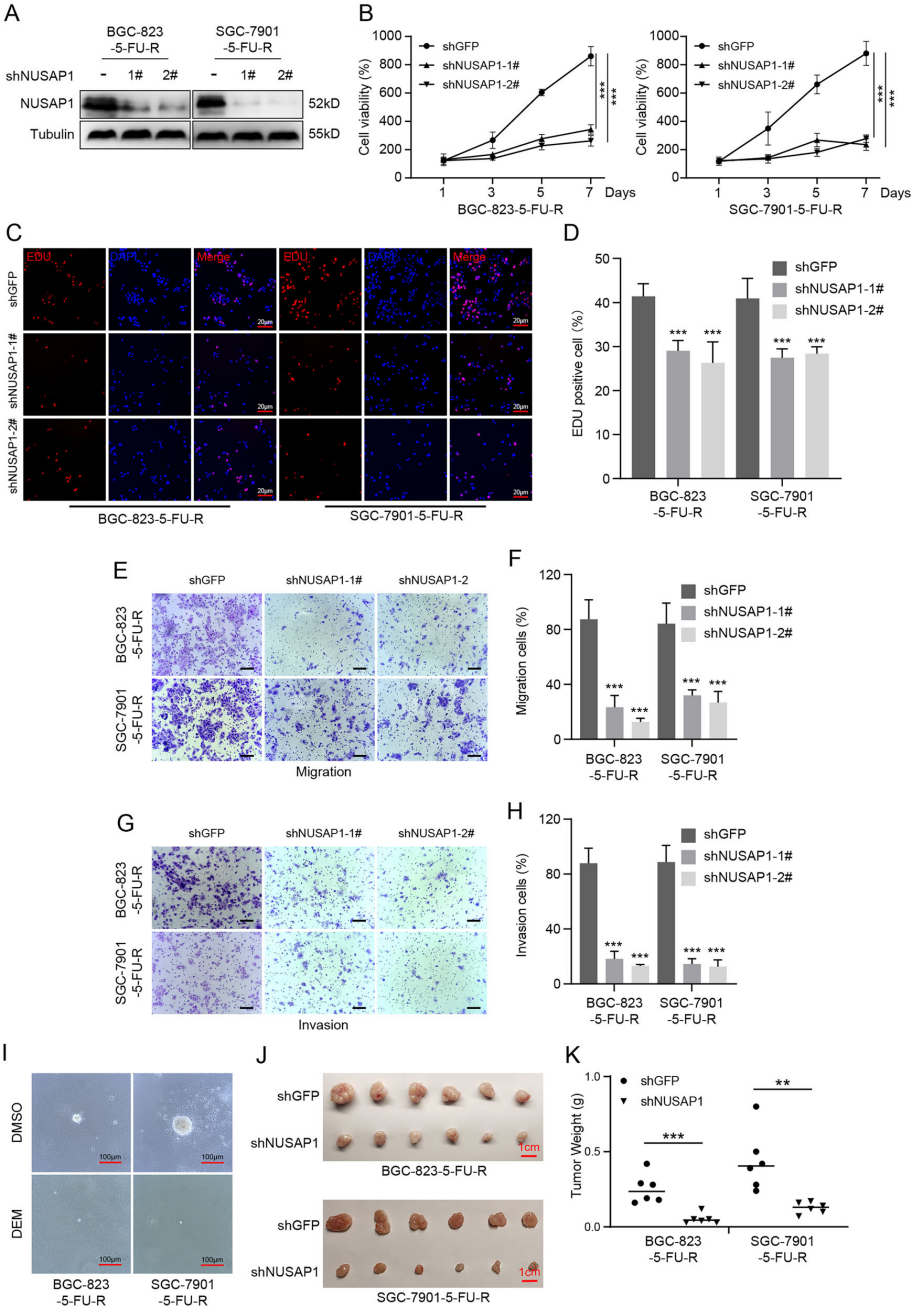
NUSAP1 promotes proliferation and tumor growth in 5-FU-resistant gastric cancer cells. **A** Western blot analysis showing NUSAP1 protein levels in BGC-823-5-FU-R and SGC-7901-5-FU-R cells transfected with shNUSAP1 (shNUSAP1-1# and shNUSAP1-2#) or shGFP control. Tubulin served as a loading control. **B** Cell viability assay performed over 7 days using BGC-823-5-FU-R and SGC-7901-5-FU-R cells transfected with shNUSAP1 or shGFP control. ***p < 0.001. **C**, **D** EdU incorporation assay showing reduced proliferation of BGC-823-5-FU-R and SGC-7901-5-FU-R cells upon NUSAP1 knockdown. Representative EdU-stained images are shown in (**C**), and the percentage of EdU-positive cells is quantified in (**D**). Scale bar = 20 μm. ***p < 0.001. **E**, **F** Transwell migration assay performed to evaluate the migratory potential of BGC-823-5-FU-R and SGC-7901-5-FU-R cells transfected with shNUSAP1 or shGFP. Representative images are shown in (**E**), and the percentage of migrated cells is quantified in (**F**). Scale bar = 100 μm. ***p < 0.001.**G, H** Transwell invasion assay showing the invasive potential of BGC-823-5-FU-R and SGC-7901-5-FU-R cells transfected with shNUSAP1 or shGFP. Representative images are shown in (**G**), and the percentage of invading cells is quantified in (**H**). Scale bar = 100 μm. ***p < 0.001. **I** Soft agar assay was conducted to assess the self-renewal ability of NUSAP1-knockdown cells compared to shGFP control. Scale bar = 100 μm. **J**, **K** In vivo subcutaneous xenograft assay in nude mice injected with NUSAP1-knockdown or control BGC-823-5-FU-R and SGC-7901-5-FU-R cells. Representative tumor images are shown in (**J**), and tumor weights are quantified in (**K**). **p < 0.01. ***p < 0.001.

To explore whether NUSAP1 affects other physiological properties of 5-FU-resistant gastric cancer cells, we examined the impact of NUSAP1 knockdown. After confirming reduced NUSAP1 expression (Fig. 2A), an MTT assay was performed to assess changes in cell proliferation rates. The results showed a significant reduction in cell viability in NUSAP1-silenced cells (Fig. 2B). Consistently, the EDU assay demonstrated a marked decrease in the percentage of EDU-positive cells in the NUSAP1-silenced group, further confirming a significant reduction in proliferation rates (Fig. 2C, D).

Previous studies have shown that 5-FU-resistant cells often exhibit enhanced migratory and invasive capabilities [10]. Therefore, we performed transwell assays to evaluate these properties. The results revealed that the migration (Fig. 2E, F) and invasion (Fig. 2G, H) capacities of NUSAP1-silenced 5-FU-resistant gastric cancer cells were significantly decreased.

To investigate whether NUSAP1 influences tumor growth in vivo, we first conducted a soft agar assay in vitro. As shown in Fig. 2I, NUSAP1 knockdown significantly reduced the size of cell colonies. Similar results were obtained in a mouse xenograft model. Equal numbers of control and NUSAP1-silenced cells were subcutaneously injected into mice, and the control group exhibited faster tumor growth, resulting in larger tumor sizes (Fig. 2J) and weights (Fig. 2K).

In summary, our results demonstrate that NUSAP1 is essential for the proliferation and tumor growth of 5-FU-resistant gastric cancer cells.

### NUSAP1 binds to PRMT1 and PRMT5

**Fig. 3:**
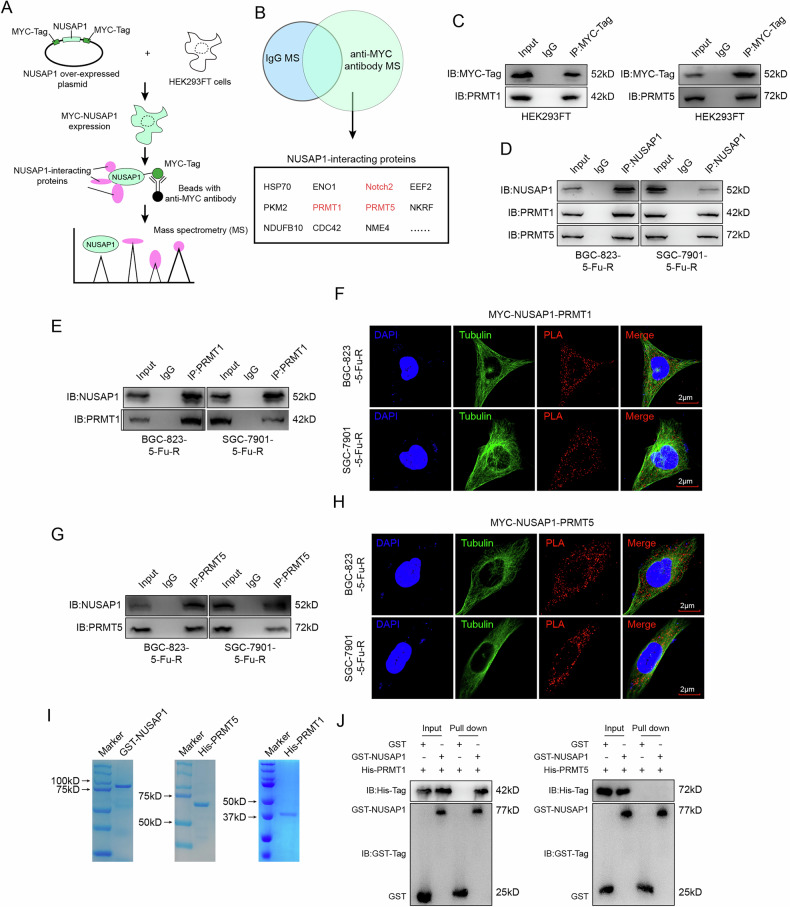
NUSAP1 Binds to PRMT1 and PRMT5. **A** Schematic representation of the experimental workflow used to identify NUSAP1-interacting proteins. HEK293FT cells were transfected with a MYC-NUSAP1 overexpression plasmid, and NUSAP1-interacting proteins were pulled down using an anti-MYC antibody followed by mass spectrometry (MS) analysis. **B** Venn diagram summarizing the NUSAP1-interacting proteins identified by MS. PRMT1 and PRMT5 were highlighted as potential interaction partners of NUSAP1. **C** Co-immunoprecipitation (Co-IP) assay performed in HEK293FT cells to confirm the interaction between NUSAP1 and PRMT1/PRMT5. MYC-tagged NUSAP1 was immunoprecipitated, and the presence of PRMT1 and PRMT5 was detected by Western blotting. **D** Co-IP assay performed in BGC-823-5-FU-R and SGC-7901-5-FU-R cells to validate the interaction between endogenous NUSAP1 and PRMT1/PRMT5. **E**, **G** Co-IP assay using anti-PRMT1 or anti-PRMT5 antibodies in BGC-823-5-FU-R and SGC-7901-5-FU-R cells to detect the presence of NUSAP1. Proximity ligation assay (PLA) showing the colocalization of NUSAP1 with PRMT1 (**F**) and PRMT5 (**H**) in BGC-823-5-FU-R and SGC-7901-5-FU-R cells. Tubulin was stained as a cytoskeletal marker. Scale bar = 2 μm. **I** Purification of GST-tagged NUSAP1, His-tagged PRMT1, and His-tagged PRMT5 proteins, as shown by SDS-PAGE. **J** GST pull-down assay was performed using GST-NUSAP1,His-PRMT1 and His-PRMT5 proteins. Pulled-down proteins were analyzed by western blotting using anti-GST and anti-His antibodies.

Previous studies have shown that NUSAP1 stabilizes substrate protein expression through interactions with other proteins [21] and forms transcriptional complexes with factors such as C-Myc and HIF-1α to regulate downstream gene expression [19]. These findings suggest that NUSAP1’s interactions with other proteins are essential for its functional roles. To identify proteins interacting with NUSAP1, we expressed MYC-tagged NUSAP1 in HEK293FT cells and performed immunoprecipitation (IP) using an anti-MYC antibody, followed by mass spectrometry analysis (Fig. 3A). As shown in Fig. 3B, multiple interacting proteins were identified, including HSP70, PKM2, Notch2, PRMT1, PRMT5, and ENO1.

Given our ongoing research on the role of arginine methylation in gastric cancer [22, 23], we analyzed the expression of PRMT1 and PRMT5 in gastric cancer patients and found that their high expression was associated with poor prognosis (Fig. S2B). Therefore, we focused on PRMT1 and PRMT5 as potential interactors of NUSAP1. Validation experiments in HEK293FT cells confirmed that MYC-NUSAP1 indeed interacts with PRMT1 and PRMT5 (Fig. 3C). To rule out interference from the MYC tag, we performed Co-immunoprecipitation (Co-IP) experiments using an anti-NUSAP1 antibody in 5-FU-resistant gastric cancer cells. The results demonstrated that NUSAP1 interacts with both PRMT1 and PRMT5 (Fig. 3D). Additionally, Co-IP experiments using antibodies against PRMT1 and PRMT5 further confirmed the presence of NUSAP1 in the precipitates of PRMT1 and PRMT5, respectively (Fig. 3E, G).

Both PRMT1 and PRMT5 are known to participate in transcriptional regulation in the nucleus and catalyze substrate methylation in the cytoplasm [24, 25]. The location of their interactions with other proteins could influence their subsequent functional roles. PLA assay was performed to determine the subcellular location of the NUSAP1-PRMT1 and NUSAP1-PRMT5 complexes. The results indicated that both complexes form predominantly in the cytoplasm (Fig. 3F, H).

To investigate whether the interactions between NUSAP1 and PRMT1/PRMT5 are direct, we expressed and purified NUSAP1, PRMT1, and PRMT5 proteins (Fig. 3I). GST pull-down assays confirmed that NUSAP1 directly binds to PRMT1, but not PRMT5 (Fig. 3J). Based on IP and PLA experiments, we found that both PRMT1 and PRMT5 interact with NUSAP1. However, GST pull-down assays revealed that PRMT5 does not directly bind to NUSAP1. Considering that NUSAP1 was unmodified in the in vitro assays, we hypothesize that post-translational modifications of NUSAP1 are required for its interaction with PRMT5.

In conclusion, we demonstrated that NUSAP1 binds to PRMT1 and PRMT5 in the cytoplasm. Based on the properties of these proteins, we hypothesize that NUSAP1 may regulate the stability of PRMT1 and PRMT5 or that PRMT1/PRMT5 may induce methylation of NUSAP1.

### PRMT1 induces asymmetric dimethylation(ADMA) of NUSAP1 at R418 and R422, but not PRMT5

**Fig. 4:**
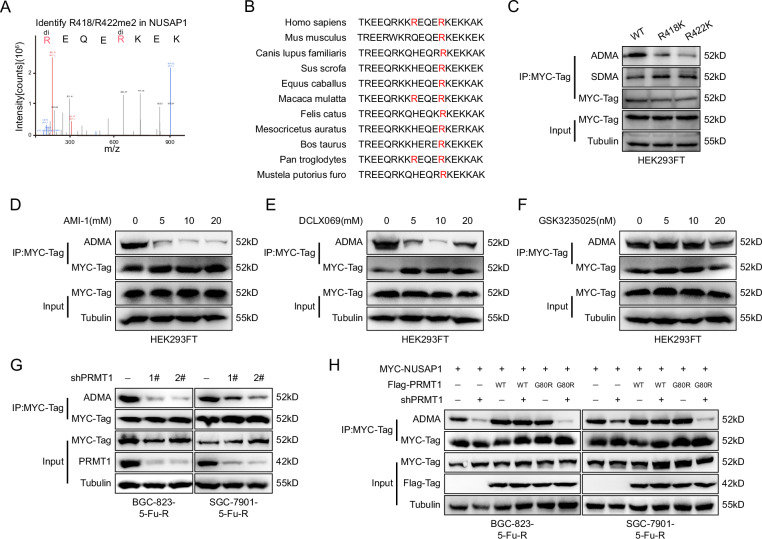
PRMT1 induces asymmetric dimethylation(ADMA) of NUSAP1 at R418 and R422, but not PRMT5. **A** Mass spectrometry (MS) analysis identifying dimethylated arginine residues (R418me2 and R422me2) on NUSAP1. **B** Sequence alignment of NUSAP1 across multiple species highlighting the conserved arginine residues (R418 and R422) subject to methylation. **C** Co-IP assay performed in HEK293FT cells expressing wild-type (WT) or mutant (R418K and R422K) MYC-tagged NUSAP1, followed by Western blotting to detect asymmetric dimethylarginine (ADMA) and symmetric dimethylarginine (SDMA). Effects of PRMT inhibitors on NUSAP1 methylation. HEK293FT cells expressing MYC-NUSAP1 were treated with increasing concentrations of PRMT1 inhibitors AMI-1 (**D**) and DCLX069 (**E**), or PRMT5 inhibitor GSK3235025 (**F**). Co-IP was performed to assess NUSAP1 methylation. **G** Co-IP assay showing reduced NUSAP1 methylation in BGC-823-5-FU-R and SGC-7901-5-FU-R cells transfected with shRNAs targeting PRMT1 (shPRMT1-1# and shPRMT1-2#) compared to control cells. **H** Co-IP assay in BGC-823-5-FU-R and SGC-7901-5-FU-R cells co-expressing wild-type (WT) or methylation-deficient mutant (G80R) Flag-PRMT1 and MYC-NUSAP1, with or without PRMT1 knockdown. Western blotting was used to detect ADMA levels on NUSAP1.

Based on previous experimental results, we investigated whether NUSAP1 protein undergoes arginine methylation. Mass spectrometry analysis identified two methylation sites, R418 and R422, on NUSAP1 (Fig. 4A). Compared to human NUSAP1 R418, the R422 site is conserved among higher eukaryotes (Fig. 4B). Further experiments revealed that mutating R418 and R422 to lysine (K) significantly reduced the levels of asymmetric dimethylation (ADMA) on NUSAP1, with no noticeable effect on symmetric dimethylation (SDMA) levels (Fig. 4C), indicating that both R418 and R422 undergo ADMA modifications.

The protein arginine methyltransferase (PRMT) family comprises nine members classified into three types based on their functions. Type I PRMTs (PRMT1, PRMT2, PRMT3, PRMT6, and PRMT8) catalyze ADMA formation, Type II PRMTs (PRMT5 and PRMT9) catalyze SDMA formation, and Type III PRMT7 exclusively catalyzes monomethylation of arginine (MMA) [25]. Since R418me2 and R422me2 are ADMA modifications, we hypothesized that these modifications are catalyzed by PRMT1.

To validate this hypothesis, cells were treated with two PRMT1 inhibitors (AMI-1 and DCLX069) and a PRMT5 inhibitor (GSK3235025). The results showed that PRMT1 inhibitors significantly reduced the methylation levels of NUSAP1(Fig. 4D, E), whereas the PRMT5 inhibitor had no effect (Fig. 4F). Additionally, silencing PRMT1 expression in cells led to a marked decrease in the ADMA levels of NUSAP1 (Fig. 4G).

Previous studies have shown that the G80 site is essential for PRMT1 enzymatic activity, and mutating G80 abolishes its methyltransferase function [26]. To further confirm the role of PRMT1, we knocked down PRMT1 in cells and subsequently overexpressed either wild-type PRMT1 (PRMT1-WT) or the G80 mutant (PRMT1-G80R). PRMT1-WT restored the ADMA levels of NUSAP1, whereas PRMT1-G80R did not (Fig. 4H).

In conclusion, our results demonstrate that PRMT1 induces asymmetric dimethylation of NUSAP1 at R418 and R422, and the G80 site of PRMT1 is crucial for its enzymatic function.

### NUSAP1 R422 promotes 5-FU resistance and cell proliferation, unlike R418

**Fig. 5:**
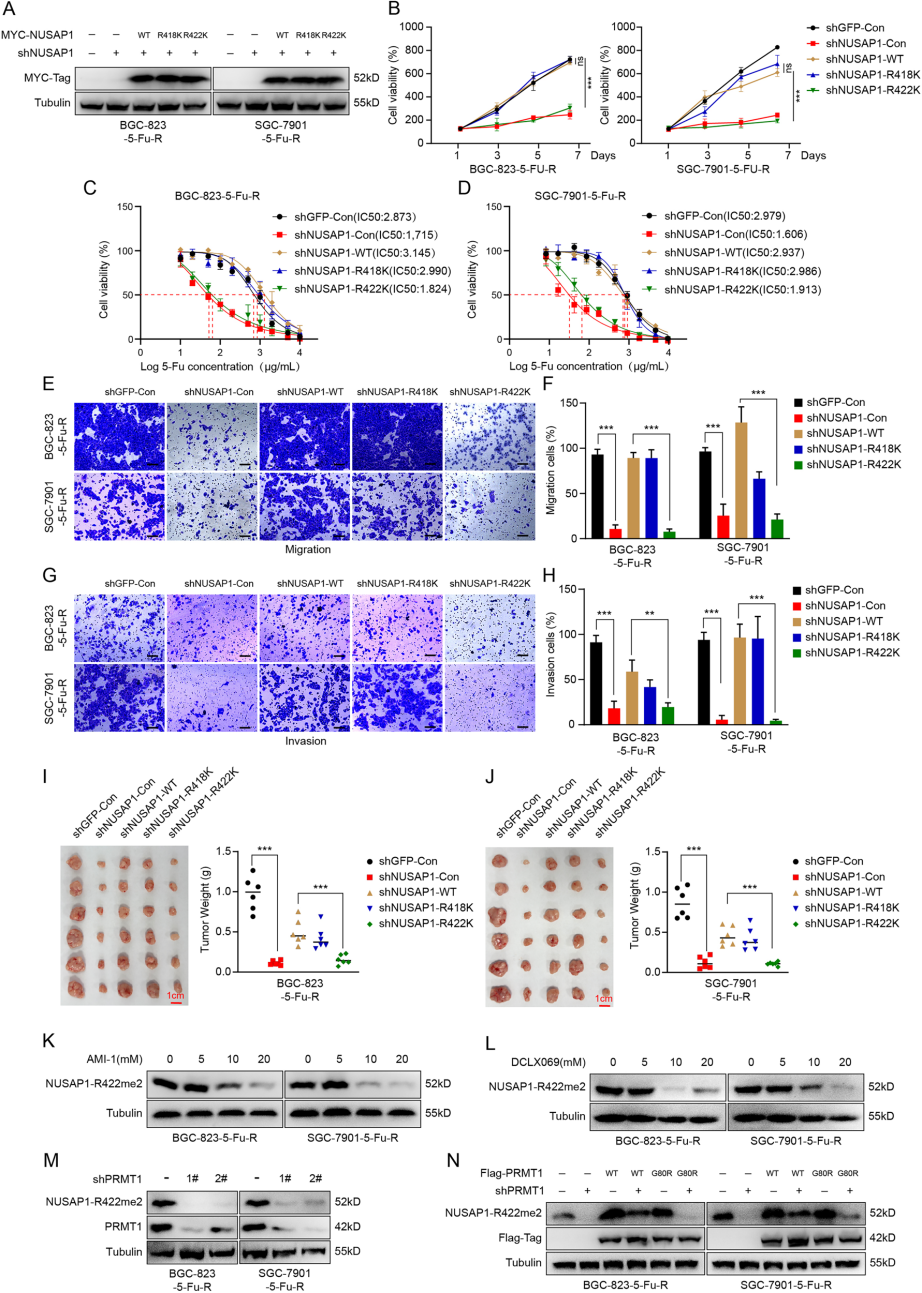
NUSAP1 R422 promotes 5-FU resistance and cell proliferation, unlike R418. **A** Western blot analysis confirming the expression of MYC-tagged wild-type (WT) NUSAP1, R418K, and R422K mutants in BGC-823-5-FU-R and SGC-7901-5-FU-R cells transfected with shNUSAP1 or control shRNA (shGFP). Tubulin was used as a loading control. **B** Cell viability assay over 7 days in NUSAP1-silenced cells expressing WT or mutant NUSAP1. ***p < 0.001. IC50 assays to evaluate the effect of WT or mutant NUSAP1 on 5-FU sensitivity in BGC-823-5-FU-R (**C**) and SGC-7901-5-FU-R (**D**) cells. Transwell migration assays showing the migratory ability of BGC-823-5-FU-R and SGC-7901-5-FU-R cells transfected with WT or mutant NUSAP1 after NUSAP1 down-regulation. Representative images are shown in (**E**), and quantitative analysis is presented in (**F**). Scale bar = 100 μm. ***p < 0.001. **G**, **H** Transwell invasion assays to assess invasive potential of cells transfected with WT or mutant NUSAP1 after NUSAP1 down-regulation. Representative images are shown in (**G**), and quantification is presented in (**H**). Scale bar = 100 μm. **p < 0.001. ***p < 0.001. **I**, **J** Subcutaneous xenograft assays in nude mice injected with cells expressing WT or mutant NUSAP1, with or without NUSAP1 knockdown. Representative tumor images and tumor weights will be shown. ***p < 0.001. Western blot analysis of R422me2 methylation levels in BGC-823-5-FU-R and SGC-7901-5-FU-R cells treated with increasing concentrations of PRMT1 inhibitors AMI-1 (**K**) and DCLX069 (**L**). Tubulin was used as a loading control. **M** Western blot analysis showing reduced R422me2 levels in BGC-823-5-FU-R and SGC-7901-5-FU-R cells transfected with shPRMT1 (shPRMT1-1# and shPRMT1-2#) compared to control. **N** Western blot analysis of R422me2 levels in cells expressing WT or methylation-deficient mutant (G80R) Flag-PRMT1 and MYC-NUSAP1, with or without PRMT1 knockdown, to confirm PRMT1 regulation of R422 methylation.

To explore the relationship between the two methylation sites of NUSAP1 (R418 and R422) and 5-FU resistance, we knocked down NUSAP1 and subsequently overexpressed wild-type NUSAP1 (NUSAP1-WT), R418 mutant (NUSAP1-R418K), and R422 mutant (NUSAP1-R422K) (Fig. 5A). As shown in Fig. 5B, cell viability assays revealed that mutation of the R422 site significantly inhibited the proliferation of 5-FU-resistant gastric cancer cells, while the R418 mutation had minimal effect. Consistently, IC50 assays demonstrated that the R422 site promotes 5-FU resistance in gastric cancer cells (Fig. 5C, D).

Additionally, transwell assays showed that overexpression of NUSAP1-R422K could not rescue the migration (Fig. 5E, F) and invasion abilities of NUSAP1-silenced cells (Fig. 5G, H). Furthermore, in mouse xenograft experiments, mutation of the R422 site abolished NUSAP1’s ability to promote tumor growth (Fig. 5I, J).

To further investigate the role of R422 methylation, we developed a specific antibody targeting NUSAP1 R422 methylation (NUSAP1 R422me2). Using this antibody, we examined its expression in both 5-FU-resistant gastric cancer cells and their parental tumor cells. The results revealed that the expression level of R422me2 was significantly higher in 5-FU-resistant cells compared to parental tumor cells (Fig. S3A). This finding suggests that R422me2 may play a crucial role in the development and maintenance of 5-FU resistance in gastric cancer. Additional, we found that R422me2 levels were significantly reduced after treatment with PRMT1 inhibitors (AMI-1 and DCLX069) (Fig. 3K, L) or PRMT1 knockdown(Fig. 3M). Moreover, re-expression of wild-type PRMT1 restored R422me2 levels, while the PRMT1 G80R mutant could not (Fig. 3N).

In summary, we found that the R422 site of NUSAP1 is critical for promoting 5-FU resistance, cell proliferation, migration, invasion, and tumor growth in gastric cancer 5-FU-resistant cells. The development of the NUSAP1 R422me2 antibody further confirmed that PRMT1 catalyzes methylation at the R422 site, underscoring its functional importance.

### NUSAP1 binds to the PEST domain of Notch2 in the cytoplasm

Studies have shown that Notch2 expression is closely associated with patient prognosis and tumor cell resistance [27–29], and our analysis further revealed that high Notch2 expression is linked to poor prognosis in gastric cancer patients (Fig. S2B). Mass spectrometry identified Notch2 as a potential NUSAP1-binding protein (Fig. 3B). Additionally, we observed a positive correlation between NUSAP1 expression and the Notch2 signaling pathway (Fig. S3B), suggesting that NUSAP1 may regulate Notch2 signaling.

**Fig. 6:**
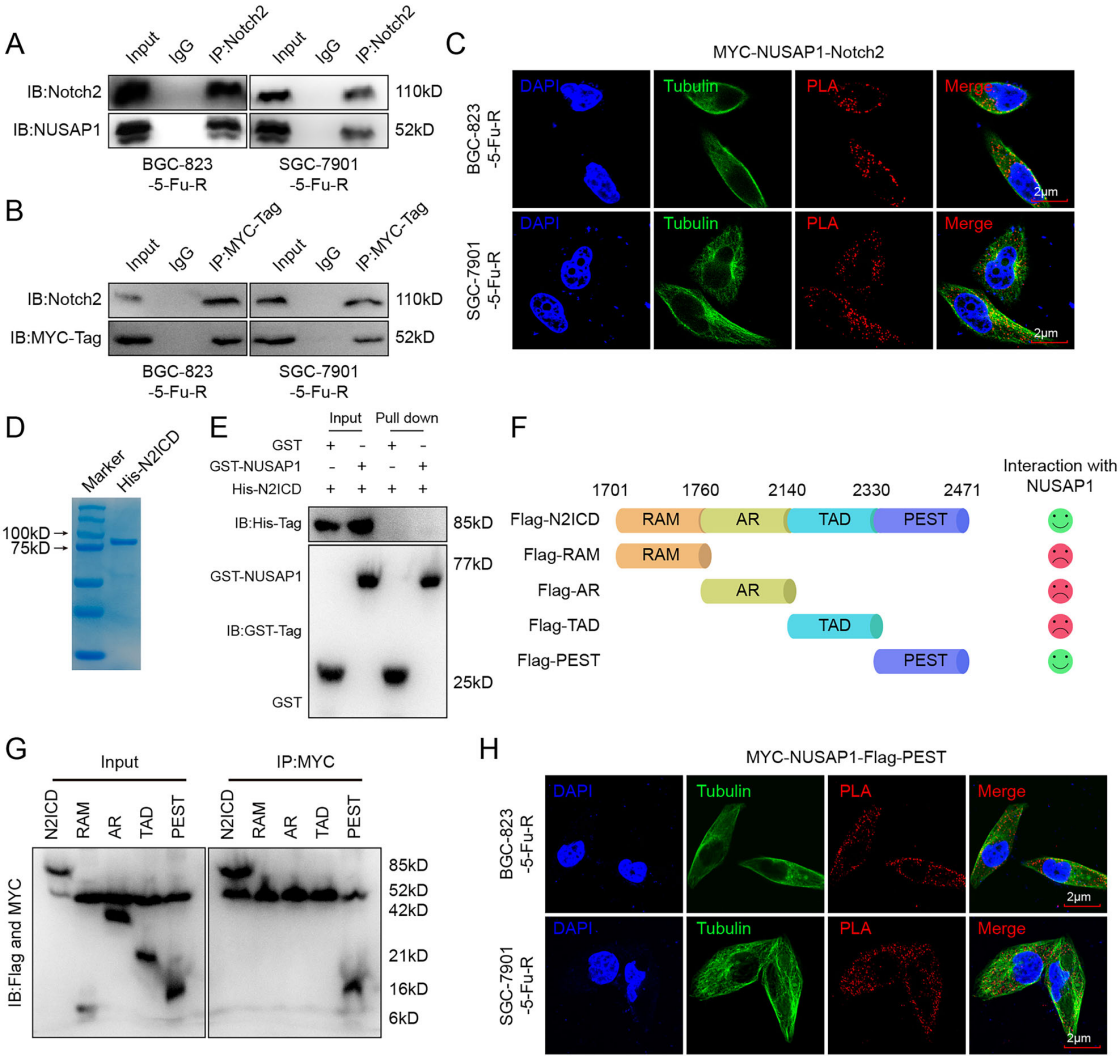
NUSAP1 binds to the PEST domain of Notch2 in the cytoplasm. **A** Co-IP assays performed in BGC-823-5-FU-R and SGC-7901-5-FU-R cells showing interaction between NUSAP1 and Notch2. Anti-Notch2 antibody was used for immunoprecipitation, and the presence of NUSAP1 was detected by Western blotting. **B** Co-IP assays using anti-MYC antibody to confirm the interaction between MYC-tagged NUSAP1 and endogenous Notch2 in BGC-823-5-FU-R and SGC-7901-5-FU-R cells. **C** PLA showing colocalization of MYC-NUSAP1 and Notch2 in BGC-823-5-FU-R and SGC-7901-5-FU-R cells. NUSAP1 and Notch2 were detected with PLA (red) and tubulin (green) as a cytoskeletal marker. Scale bar = 2 μm. **D** SDS-PAGE showing the purification of His-tagged Notch2 intracellular domain (N2ICD) protein. **E** GST pull-down assay was performed using GST-NUSAP1 and His-tagged N2ICD proteins. Pulled-down proteins were analyzed by Western blotting using anti-GST and anti-His antibodies. **F** Diagram of the Notch2 intracellular domain (N2ICD) with functional regions: RAM, AR, TAD, and PEST. Interaction of these regions with NUSAP1 was assessed and is indicated by colored circles. **G** Co-IP assays in BGC-823-5-FU-R and SGC-7901-5-FU-R cells showing that the PEST domain of Notch2 interacts with NUSAP1. Flag-tagged N2ICD fragments (RAM, AR, TAD, PEST) were immunoprecipitated using anti-MYC antibody, and NUSAP1 was detected by Western blotting. **H** PLA showing colocalization of MYC-NUSAP1 and Flag-PEST domain in BGC-823-5-FU-R and SGC-7901-5-FU-R cells. Scale bar = 2 μm.

First, Co-IP experiments using anti-Notch2 antibody in BGC-823-5-FU-R and SGC-7901-5-FU-R cells revealed that NUSAP1 interacts with Notch2 (Fig. 6A). Moreover, Co-IP experiments using anti-MYC antibodies in 5-FU-resistant cells overexpressing MYC-NUSAP1 confirmed the presence of Notch2 in the precipitates (Fig. 6B). PLA assay further demonstrated that the interaction between NUSAP1 and Notch2 occurs in the cytoplasm (Fig. 6C), suggesting that NUSAP1 does not form a transcriptional complex with Notch2 to regulate Notch2 signaling pathway.

Since Notch2 is cleaved into an extracellular domain (N2ECD) and an intracellular domain (N2ICD), we next investigated whether N2ICD directly interacts with NUSAP1. Purified N2ICD protein (Fig. 6D) was used in GST pull-down assays. Surprisingly, we found that N2ICD does not directly interact with NUSAP1 (Fig. 6E). N2ICD consists of four domains: the RBPjκ association module (RAM), ankyrin repeat domain (AR), transactivation domain (TAD), and proline/glutamic acid/serine/threonine-rich motifs (PEST). Determining the specific domain involved in the NUSAP1-N2ICD interaction is crucial for understanding its functional impact. To identify the binding domain, we constructed Flag-tagged vectors for each domain (Fig. 6F). Co-IP experiments revealed that NUSAP1 specifically binds to the PEST domain of N2ICD (Fig. 6G), a finding further validated by PLA analysis (Fig. 6H).

These results demonstrate that NUSAP1 binds to the PEST domain of N2ICD in the cytoplasm, suggesting that this interaction may mediate NUSAP1’s involvement in the regulation of the Notch2 signaling pathway.

### NUSAP1 stabilizes Notch2 expression by binding to N2ICD through the R422 site

**Fig. 7:**
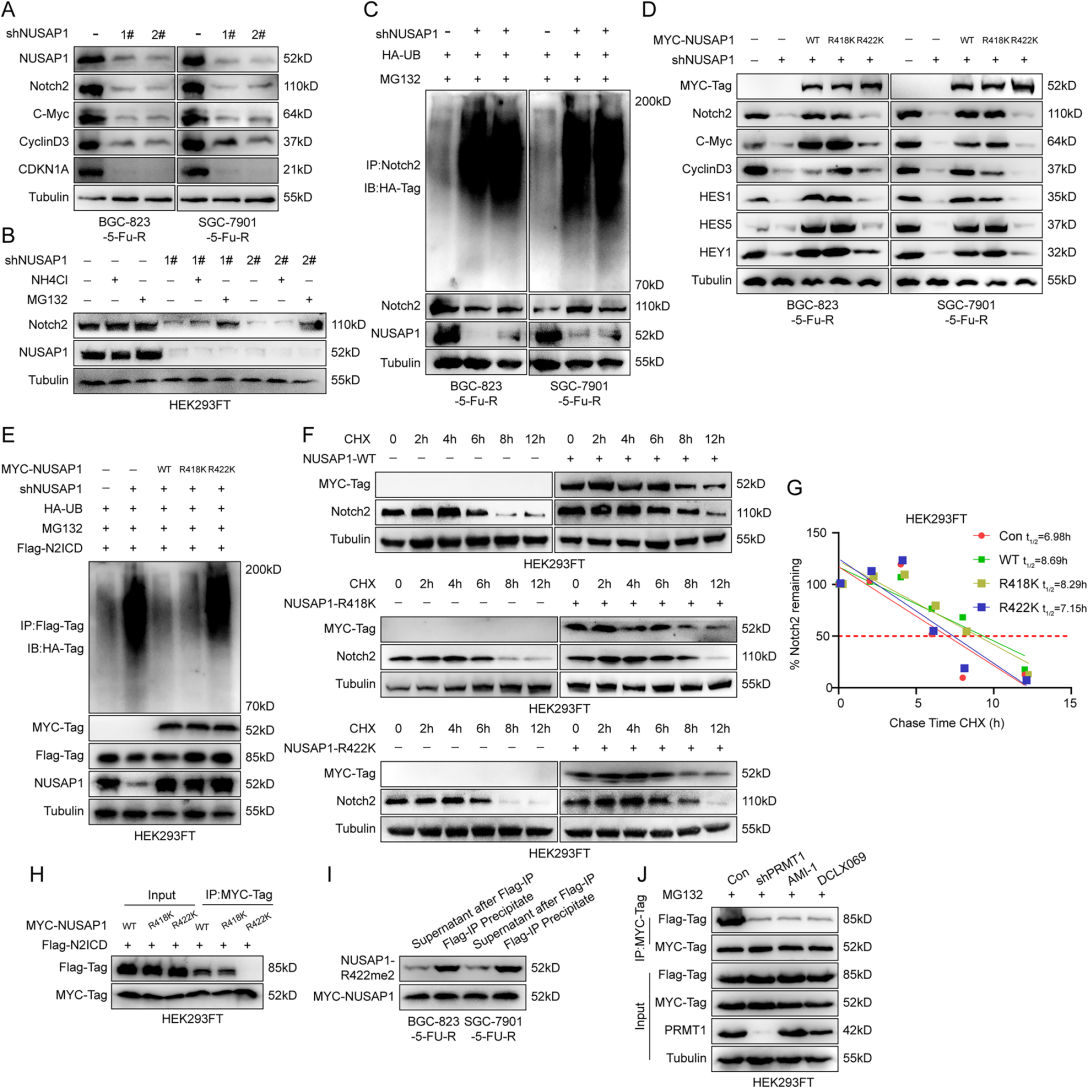
NUSAP1 stabilizes Notch2 expression by binding to N2ICD through the R422 site. **A** Western blot analysis showing the effects of NUSAP1 knockdown on Notch2 and its downstream signaling proteins (C-Myc, CyclinD3 and CDKN1A) in BGC-823-5-FU-R and SGC-7901-5-FU-R cells. Tubulin was used as a loading control. **B** Western blot analysis of NUSAP1 and Notch2 protein levels in HEK293FT cells treated with NH_4_Cl or MG132, with or without NUSAP1 knockdown. **C** Co-IP assay in BGC-823-5-FU-R and SGC-7901-5-FU-R cells showing that NUSAP1 knockdown promotes Notch2 ubiquitination. HA-tagged ubiquitin (HA-Ub) was used to detect ubiquitinated Notch2 in the presence of MG132. **D** Western blot analysis of Notch2 and its downstream signaling proteins in cells expressing MYC-tagged wild-type (WT) or mutant (R418K, R422K) NUSAP1, demonstrating the importance of R422 methylation for regulating Notch2 signaling. **E** Co-IP assay showing that WT NUSAP1 and R418K NUSAP1, but not the R422K mutant, inhibits ubiquitination of Flag-tagged N2ICD in HEK293FT cells treated with MG132. **F**, **G** Cycloheximide (CHX) chase assay to determine the half-life of Notch2 in HEK293FT cells expressing WT or mutant (R418K, R422K) NUSAP1. Western blot analysis of Notch2 protein levels over time is shown, with degradation kinetics plotted in (**G**). **H** Co-IP assay showing the binding between MYC-NUSAP1 WT, R418K, R422K and Flag-N2ICD in HEK293FT cells. **I** Western blot analysis using the NUSAP1-R422me2-specific antibody showing that R422 methylation enables NUSAP1 to bind to Flag-tagged N2ICD in BGC-823-5-FU-R and SGC-7901-5-FU-R cells. **J** Western blot analysis showing the effects of PRMT1 inhibitors (AMI-1 and DCLX069) and PRMT1 down-regulation on the interaction between MYC-NUSAP1 and Flag-N2ICD.

Since the PEST domain stabilizes N2ICD expression, we hypothesized that NUSAP1 might regulate the stability of Notch2 protein. To test this, we first examined changes in the Notch2 signaling pathway after silencing NUSAP1. As shown in Fig. 7A, silencing NUSAP1 inhibited Notch2 signaling. Protein degradation can occur through autophagy-dependent or ubiquitination-dependent pathways [30, 31]. After silencing NUSAP1, cells were treated with NH_4_Cl (autophagy inhibitor) or MG132 (proteasome inhibitor). MG132 treatment significantly mitigated NUSAP1 knockdown-induced Notch2 degradation (Fig. 7B). Ubiquitination assays further revealed that the ubiquitination level of Notch2 increased significantly in NUSAP1-silenced cells (Fig. 7C), suggesting that NUSAP1 inhibits Notch2 ubiquitination and subsequent degradation.

Given these findings, we questioned whether NUSAP1 methylation is critical for its regulation of Notch2 ubiquitination. To address this, we expressed NUSAP1-WT, NUSAP1-R418K, and NUSAP1-R422K in NUSAP1-silenced cells. Mutation at the R422 site failed to rescue the suppression of Notch2 signaling caused by NUSAP1 knockdown, whereas R418 mutation had no such effect (Fig. 7D). Additionally, analysis using the GEPIA2.0 database revealed a significant positive correlation between Notch2 signaling pathway activity and PRMT1 expression (Fig. S3C). Moreover, silencing or inhibiting PRMT1, which catalyzes R422 methylation, also suppressed Notch2 signaling (Fig. S3D). Ubiquitination assays showed that in cells expressing NUSAP1-R422K, the ubiquitination level of N2ICD remained unchanged compared to NUSAP1-silenced cells (Fig. 7E). Furthermore, while overexpression of NUSAP1-WT or NUSAP1-R418K extended the half-life of Notch2, NUSAP1-R422K did not (Fig. 7F, G), indicating the importance of the R422 site for NUSAP1’s function.

Arginine methylation influences various physiological activities, such as protein localization, stability, transcriptional activity, and binding capacity [32, 33]. To investigate the effect of the R422 site on NUSAP1 function, we performed immunofluorescence (IF) and half-life assays. Mutation of R422 had no significant impact on NUSAP1 subcellular localization (Fig. S3E, F) or half-life (Fig. S3G, H). However, Co-IP experiments revealed that NUSAP1-R422K failed to co-precipitate with N2ICD (Fig. 7H).

To investigate whether N2ICD preferentially binds to R422-methylated NUSAP1, we performed Co-IP assays using Flag-tagged N2ICD and Myc-tagged NUSAP1. After immunoprecipitation with anti-Flag antibody, the methylation level of NUSAP1 (R422me2) was detected in the cell lysate (supernatant) and the immunoprecipitated complex (precipitate). As shown in Fig. 7I, the R422me2 level was significantly higher in the Flag-IP precipitate than in the supernatant, indicating that N2ICD preferentially binds R422-methylated NUSAP1. Additionally, PRMT1 knockdown or inhibition significantly reduced the interaction between N2ICD and NUSAP1 (Fig. 7J). This indicates that only NUSAP1 methylated by PRMT1 can interact with proteins such as Notch2. This also explains why the interactions between NUSAP1, PRMT5, and Notch2 can be detected through IP and PLA, but not through GST pull-down assays. We also attempted to directly use the NUSAP1 R422me2-specific antibody for IP experiments. Unfortunately, while the antibody performed well in Western blotting (WB) assays, it was not effective in IP experiments (Fig. S3I).

In conclusion, NUSAP1 binds to N2ICD through its R422 site, inhibiting N2ICD ubiquitination and degradation, thereby stabilizing Notch2 expression.

## Discussion

5-Fluorouracil (5-FU) has been utilized as a clinical chemotherapy agent for over 60 years, showing efficacy in treating various cancers [3]. However, the emergence of 5-FU resistance in many patients significantly diminishes its therapeutic effectiveness. Understanding the mechanisms underlying 5-FU resistance to enhance its efficacy and counteract resistance has become a major challenge in clinical oncology. Several mechanisms have been reported to contribute to 5-FU resistance, such as tumor stem cells within the tumor microenvironment (TME), which reduce the sensitivity of cancer cells to 5-FU [8, 9]. Additionally, the expression of stem cell markers (e.g., Notch, CD44, ALDHA1, Oct4, and Sox2) directly enhances resistance to 5-FU, and resistant cells often exhibit increased migratory and invasive capabilities [10]. Moreover, p53 loss of expression [34] and hyperactivation of the RAS/PI3K/AKT signaling pathway [35] have also been linked to 5-FU resistance.

In this study, we identified NUSAP1 as a key regulator of 5-FU resistance. Through proteomic analysis combined with in vitro and in vivo experiments, we found that NUSAP1 is highly expressed in 5-FU-resistant gastric cancer cells, enhancing their resistance to 5-FU (Fig. 1) and promoting their proliferation, migration, and tumor growth (Fig. 2).

NUSAP1 exhibits different functions depending on its subcellular localization. In the cytoplasm, it interacts with substrate proteins to regulate their stability [15, 17]. In the nucleus, NUSAP1 forms transcriptional complexes with factors such as HIF1α and C-Myc to regulate downstream gene expression [19]. To explore the functional mechanism of NUSAP1, we identified its interacting proteins, including PRMT1, PRMT5, and Notch2 (Fig. 3). Both PRMT1 and PRMT5, members of the protein arginine methyltransferase (PRMT) family, play crucial roles in tumor development. In the nucleus, they form transcriptional complexes with various factors, regulating gene expression. For instance, PRMT1-mediated H4R3 methylation enhances protein synthesis, promoting leukemia cell self-renewal [36] and activating the EGFR signaling pathway [37]. PRMT5 also methylates H4R3, contributing to chromatin regulation [38]. Additionally, both PRMT1 and PRMT5 methylate several non-histone substrates, such as BRD4 [39], STAT3 [40], and SMYD4 [41], thereby amplifying their functional roles. Notably, both enzymes are implicated in chemoresistance, and targeting PRMT1 and PRMT5 has demonstrated therapeutic potential in various cancers [42, 43]. Our previous studies have shown that PRMT1 and PRMT4/CARM1 promote gastric cancer proliferation and metastasis [22, 23], underscoring the significance of NUSAP1 interactions with PRMT1 and PRMT5.

We observed that NUSAP1 binds to PRMT1 and PRMT5 in the cytoplasm (Fig. 3), suggesting that the NUSAP1-PRMT1 and NUSAP1-PRMT5 complexes are not involved in transcriptional regulation. Instead, NUSAP1 may regulate the stability of PRMT1 and PRMT5, or conversely, PRMT1 and PRMT5 may methylate NUSAP1. Mass spectrometry analysis revealed arginine methylation at R418 and R422 of NUSAP1, both catalyzed by PRMT1 (Fig. 4). Functional assays demonstrated that R422 methylation, but not R418, is critical for 5-FU resistance and cell proliferation (Fig. 5). Using a specific R422me2 antibody, we confirmed that R422 methylation is highly expressed in resistant cells, further emphasizing its functional role. However, the current R422me2 antibody cannot be used for immunoprecipitation, necessitating further optimization for more in-depth studies.

The Notch signaling pathway, discovered over a century ago, has been extensively studied for its pivotal role in cell fate determination [44, 45]. Notch signaling is implicated in tumorigenesis and chemoresistance [46–48]. Notch2, a key component of the Notch pathway, regulates oncogenes such as Myc, CDKN1A, and Cyclin D3 [49]. Post-translational modifications (e.g., methylation, glycosylation, and phosphorylation) of Notch2 affect its localization and ligand binding, influencing Notch2 signaling [22, 50, 51]. Investigating the epigenetic modifications of Notch2 and their effects on Notch2 signaling could provide insights into overcoming chemoresistance.

We found that NUSAP1 interacts with the PEST domain of Notch2, which regulates Notch2 protein stability (Fig. 6). NUSAP1 inhibits Notch2 ubiquitination and degradation, stabilizing Notch2 protein (Fig. 7). Importantly, this interaction depends on R422 methylation of NUSAP1. Stabilized Notch2 activates downstream signaling components such as C-Myc, Cyclin D3, and HES1, which drive cancer cell survival and chemoresistance. Our findings suggest that targeting the NUSAP1-Notch2 axis could be an effective strategy to reverse 5-FU resistance.

In conclusion, our study identifies a novel mechanism by which PRMT1-mediated methylation of NUSAP1 promotes 5-FU resistance in gastric cancer by stabilizing Notch2. The NUSAP1-Notch2 axis represents a promising therapeutic target for overcoming chemoresistance in gastric cancer. These findings highlight the critical role of post-translational modifications in regulating protein stability and signaling pathways in cancer biology. Future studies should validate these findings in clinical settings and explore therapeutic strategies targeting this axis.

## Supplementary information

Supplementary information

Original western blot for Figure1-3

Original western blot for Figure4

Original western blot for Figure5-6

Original western blot for Figure7

Original western blot for FigureS1-S3

## Data Availability

The datasets generated and/or analyzed during the current study are not publicly available but are available from the corresponding author on reasonable request.
