# Supplementary material for: PRMT1-catalyzed NUSAP1 methylation enhances Notch2 signaling and 5-FU resistance in gastric cancer
Source: Cell Death Dis. 2025 May 20;16(1):404. doi: 10.1038/s41419-025-07723-9 (PMC12092681; doi:10.1038/s41419-025-07723-9)
Supplement: Supplementary file 1 — Supplementary information [file 41419_2025_7723_MOESM1_ESM.pdf]

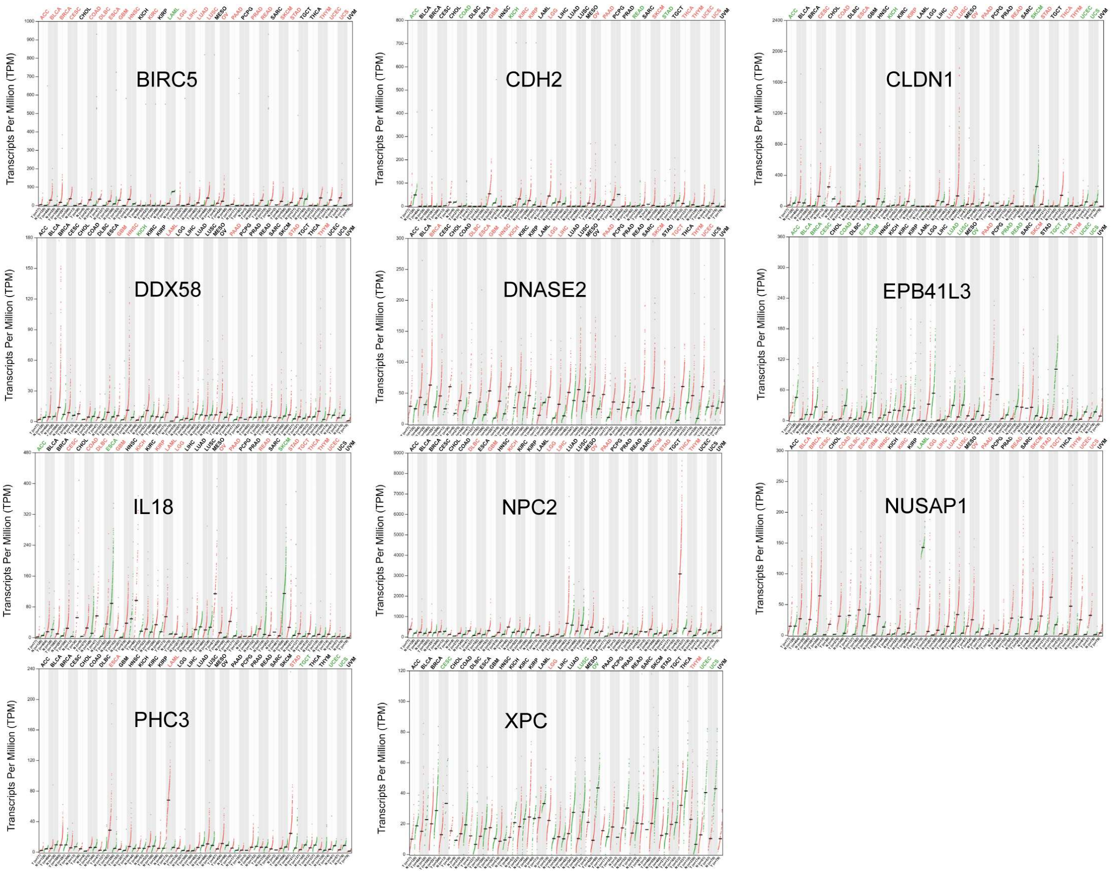

Fig.S1 Expression analysis of 11 commonly upregulated proteins in various tumors using the GEPIA2 database. Transcript per million (TPM) expression levels of BIRC5, CDH2, CLDN1, DDX58, DNASE2, EPB41L3, IL18, NPC2, NUSAP1, PHC3, and XPC were analyzed across various tumor types and corresponding normal tissues using the GEPIA2 database. The red points represent genes significantly upregulated in tumors, while green points indicate genes downregulated in tumors compared to normal tissues.

A

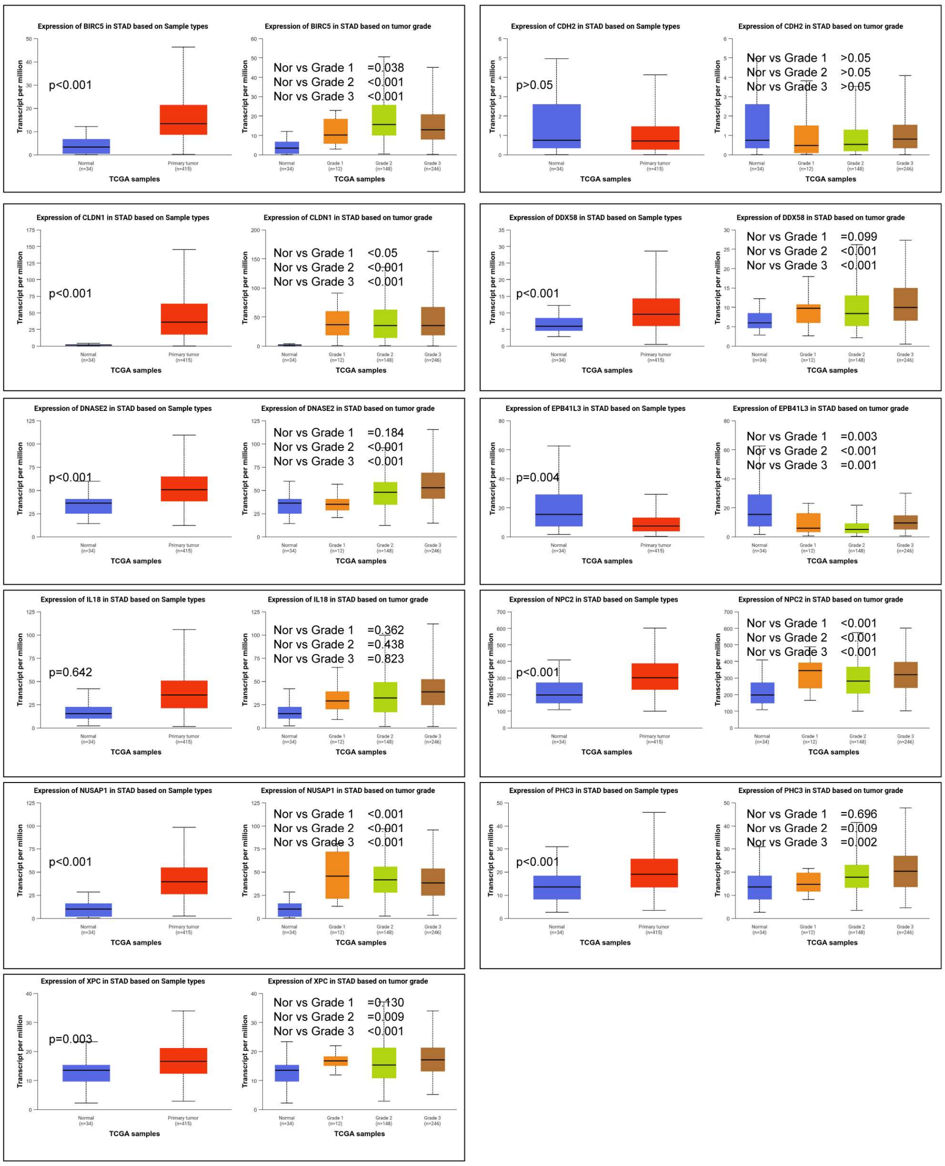

B

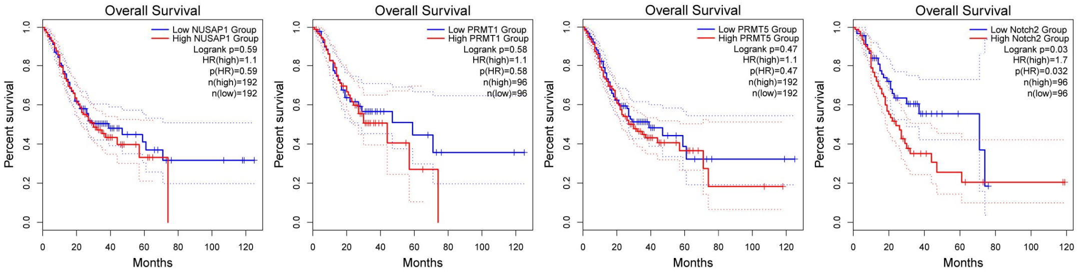

Fig.S2 Analysis of the expression levels of 11 commonly upregulated proteins and their correlation with tumor grade in gastric cancer using the TCGA database. A Boxplots showing the expression levels (TPM) of BIRC5, CDH2, CLDN1, DDX58, DNASE2, EPB41L3, IL18, NPC2, NUSAP1, PHC3, and XPC in normal tissues (Nor) and gastric cancer (STAD) samples, as well as their association with tumor grades (Grade 1, Grade 2, and Grade 3). Statistical significance between normal and tumor samples, as well as between different tumor grades, is indicated (p-values). **B** The Relationship Between Multiple Genes （NUSAP1,PRMT1,PRMT5 and Notch2）and Gastric Cancer Prognosis.

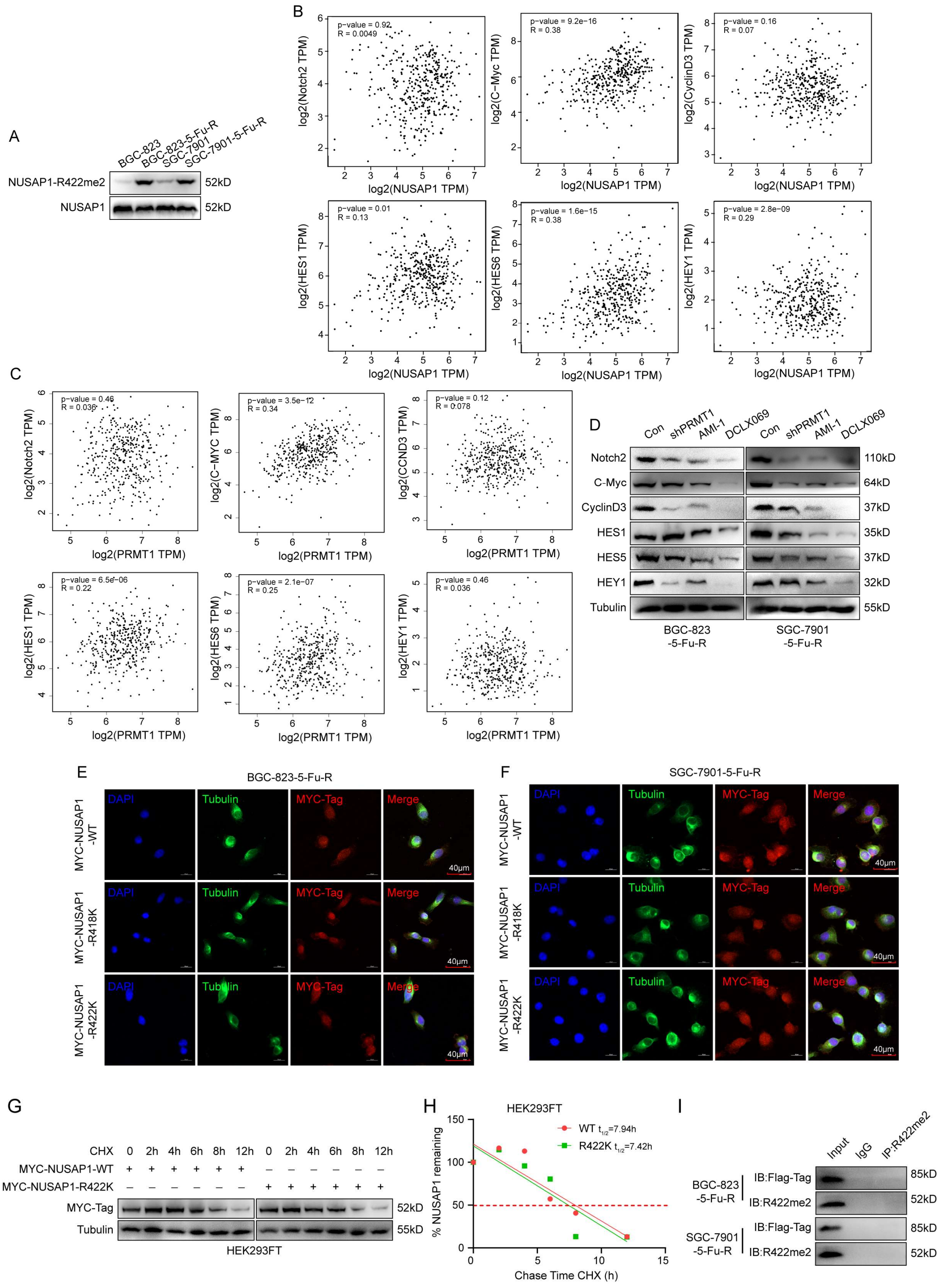

Fig.S3 Analysis of NUSAP1 R422me2 expression and its functional role in Notch2 signaling, subcellular localization, and protein stability. A Western blot analysis of NUSAP1 R422me2 levels in parental gastric cancer cells and their corresponding 5-FU-resistant cell lines (BGC-823 and SGC-7901). **B** Correlation analysis between NUSAP1 expression and Notch2 signaling components using GEPIA2.0 database. Pearson correlation coefficients (R) and p-values are shown. **C** Correlation analysis between PRMT1 expression and Notch2 signaling components using GEPIA2.0 database. Pearson correlation coefficients (R) and p-values are shown. **D** Western blot analysis of Notch2 and its downstream effectors

(C-Myc, CyclinD3, HES1, HES5, HEY1) in BGC-823-5-FU-R and SGC-7901-5-FU-R cells with PRMT1 knockdown (shPRMT1) or PRMT1 inhibitors treatment (AMI-1 and DCLX069). **E-F** Immunofluorescence (IF) staining showing the subcellular localization of MYC-tagged WT NUSAP1 and mutants (R418K and R422K) in BGC-823-5-FU-R (**E**) and SGC-7901-5-FU-R (**F**) cells. Tubulin was used as a cytoskeletal marker. Scale bar = 40 μm. **G** Western blot analysis of NUSAP1 protein levels in HEK293FT cells expressing WT or R422K NUSAP1 at different time points after cycloheximide (CHX) treatment to assess protein stability. **H** Degradation kinetics of WT and R422K NUSAP1 in HEK293FT cells based on CHX chase assay. Protein half-life ($t_1/_2$) was calculated and indicated. **I** IP assay using R422me2-specific antibody to detect the interaction of R422-methylated NUSAP1 with Flag-tagged Notch2 intracellular domain (N2ICD) in BGC-823-5-FU-R and SGC-7901-5-FU-R cells.
