# Supplementary figures and images for: PRMT1-catalyzed NUSAP1 methylation enhances Notch2 signaling and 5-FU resistance in gastric cancer
Source: Cell Death Dis. 2025 May 20;16(1):404. doi: 10.1038/s41419-025-07723-9 (PMC12092681; doi:10.1038/s41419-025-07723-9)

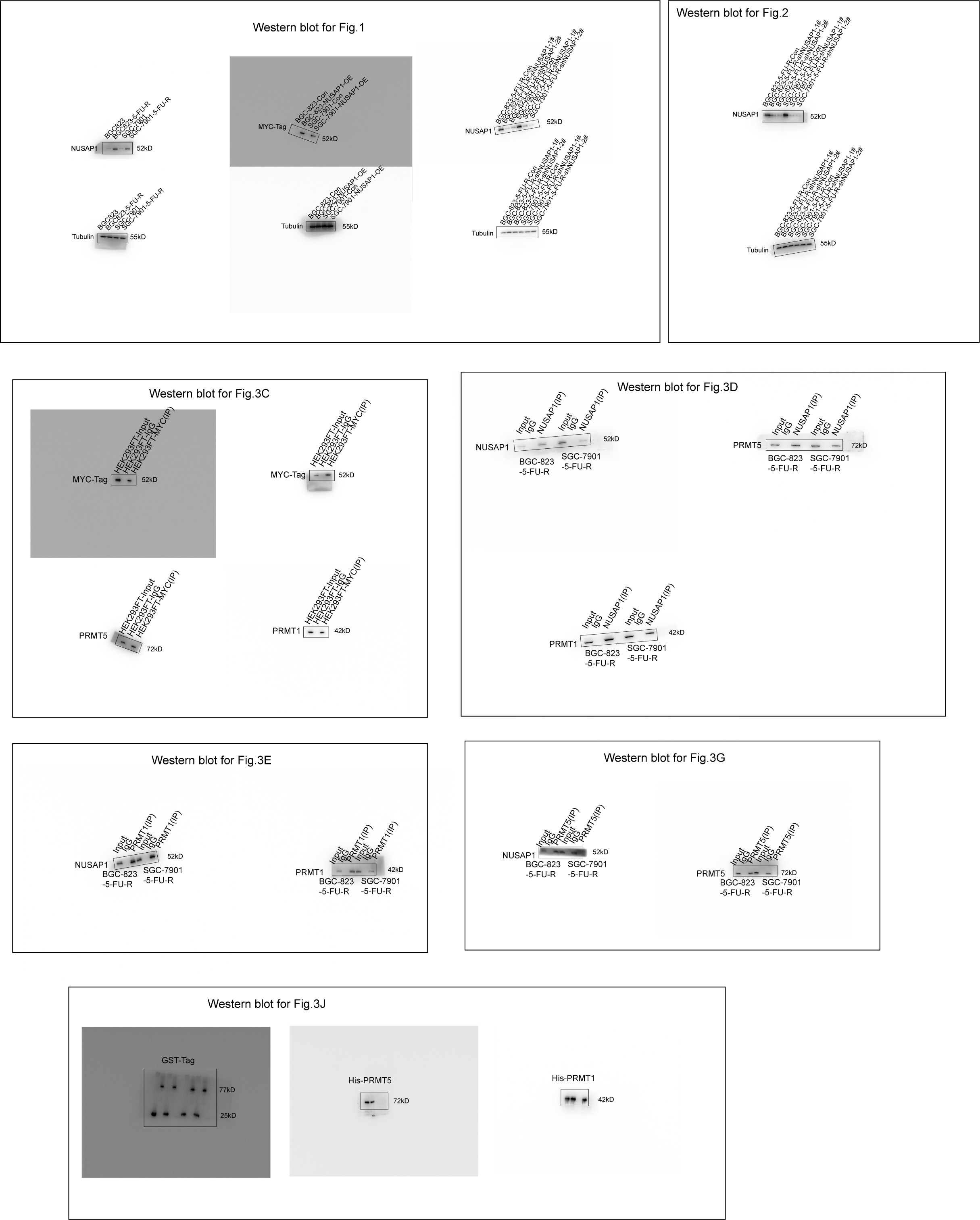

Supplement: Supplementary file 2 — Original western blot for Figure1-3 [file 41419_2025_7723_MOESM2_ESM.tif]

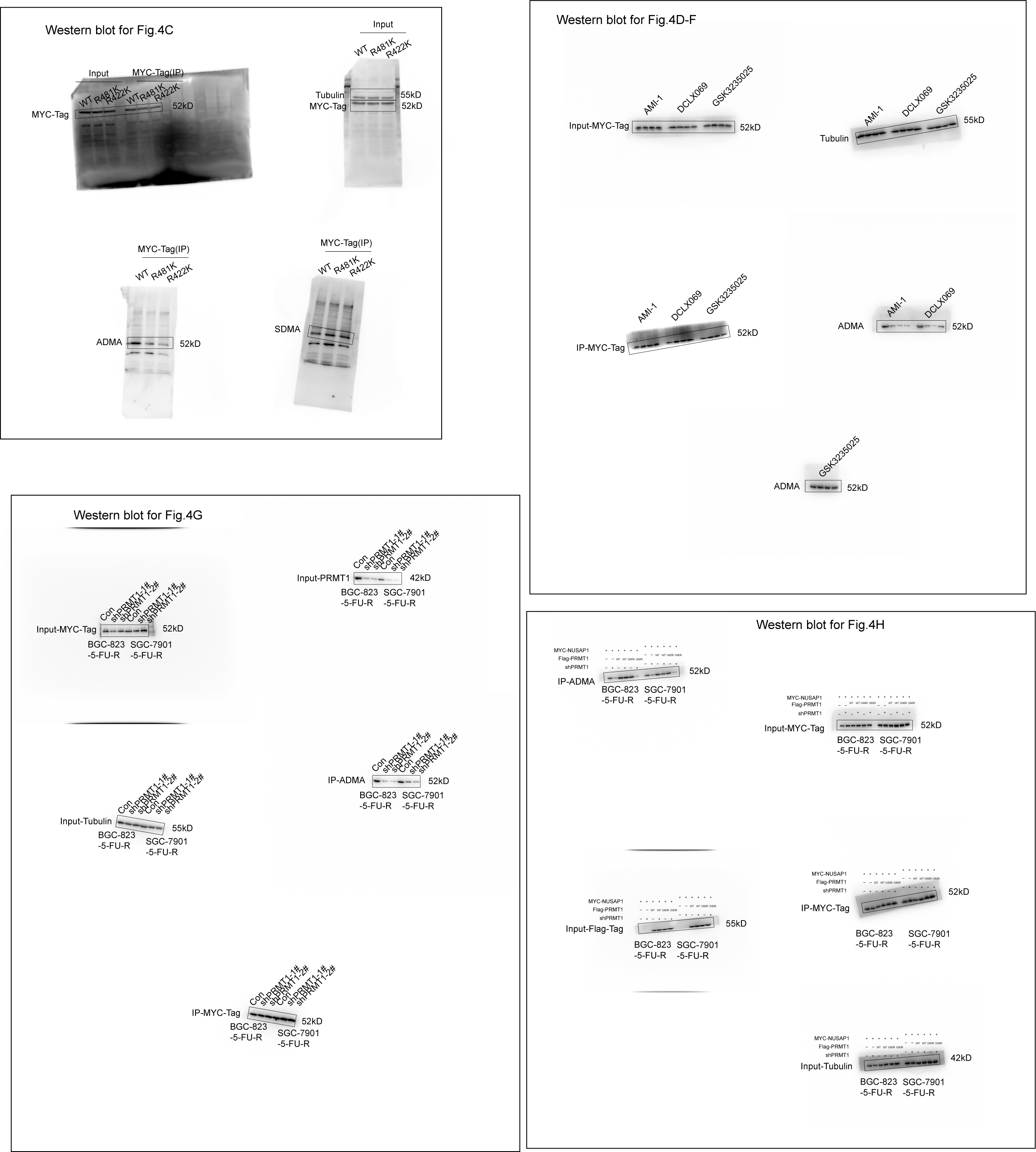

Supplement: Supplementary file 3 — Original western blot for Figure4 [file 41419_2025_7723_MOESM3_ESM.tif]

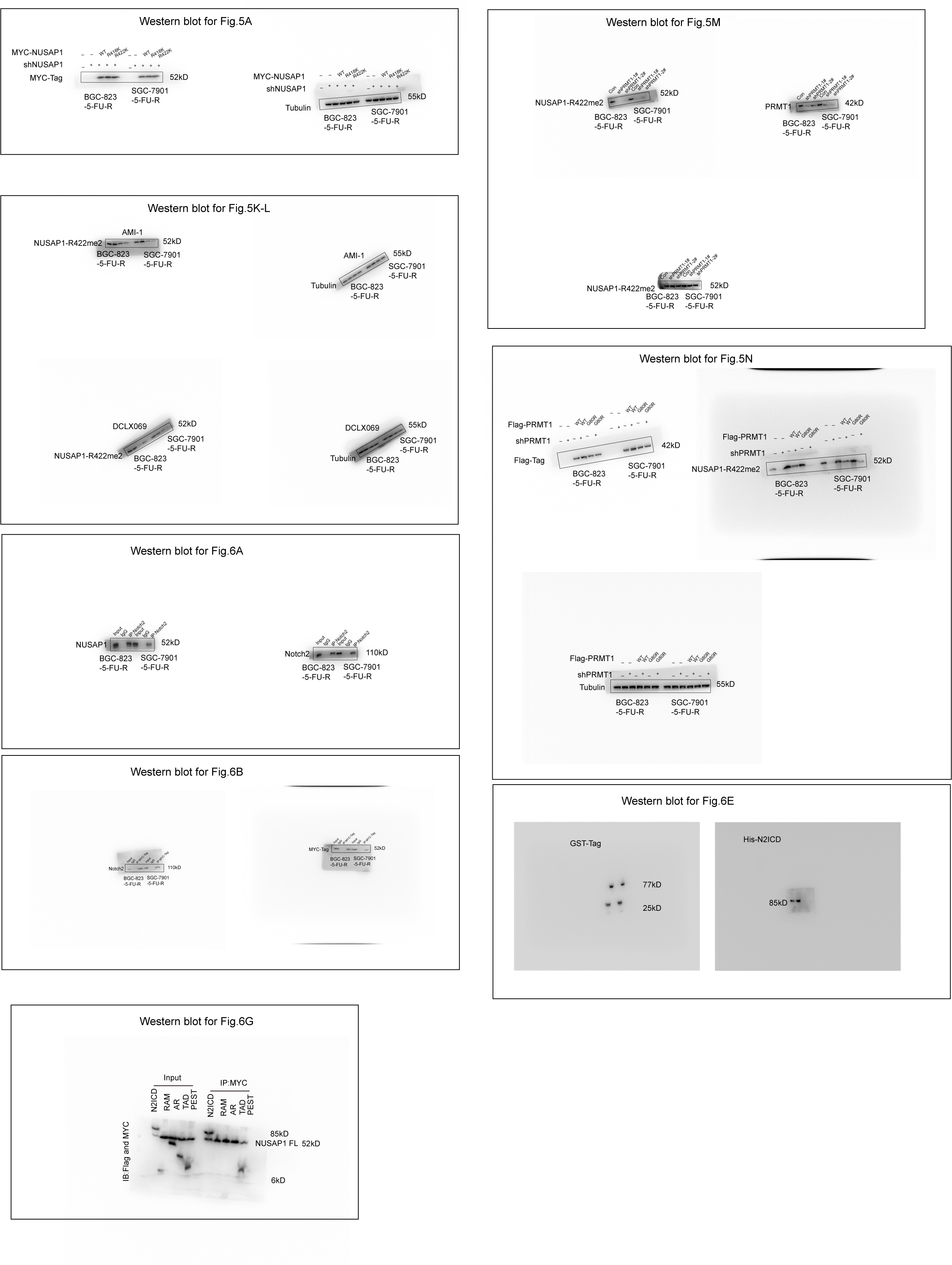

Supplement: Supplementary file 4 — Original western blot for Figure5-6 [file 41419_2025_7723_MOESM4_ESM.tif]

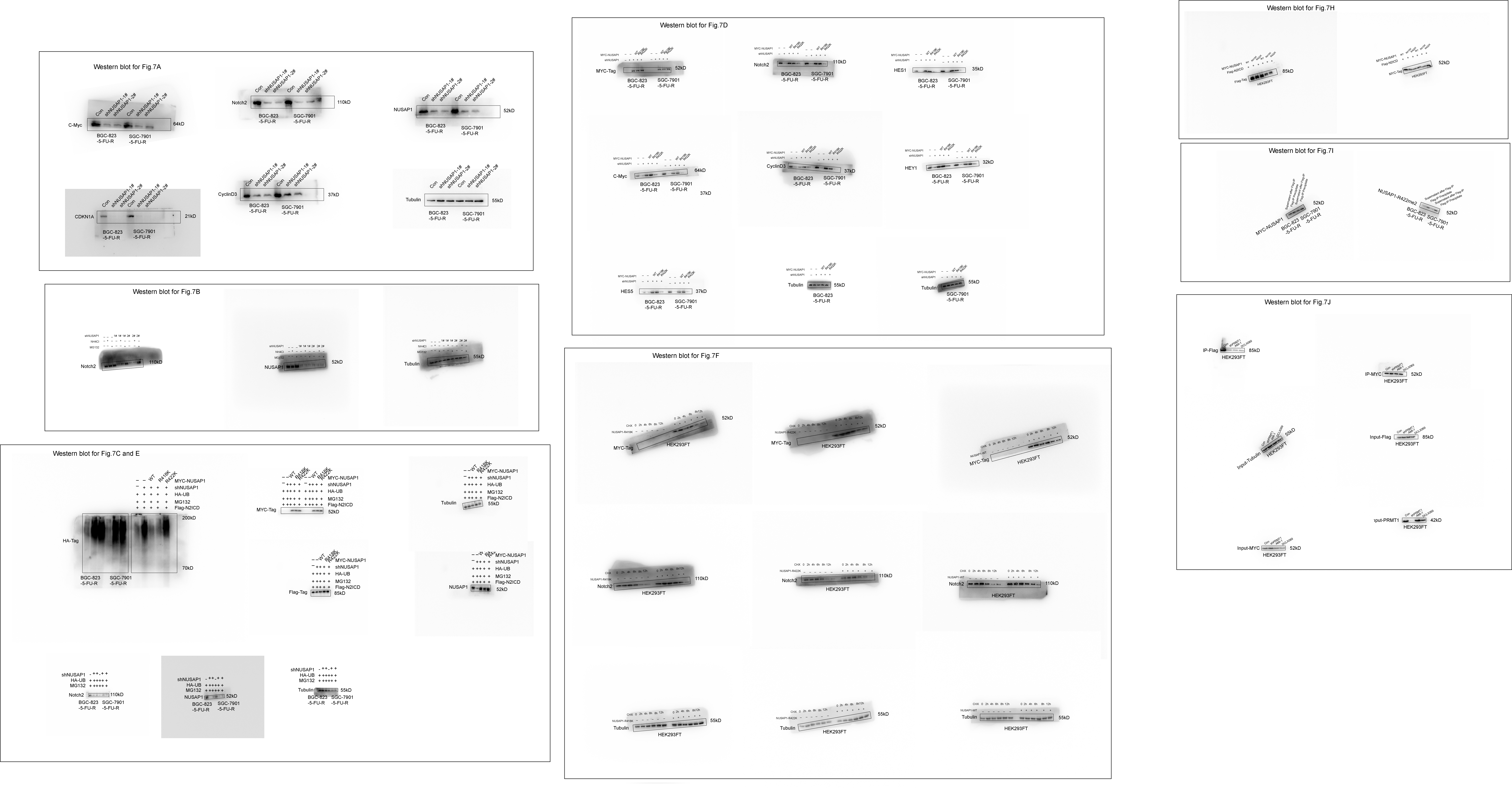

Supplement: Supplementary file 5 — Original western blot for Figure7 [file 41419_2025_7723_MOESM5_ESM.tif]

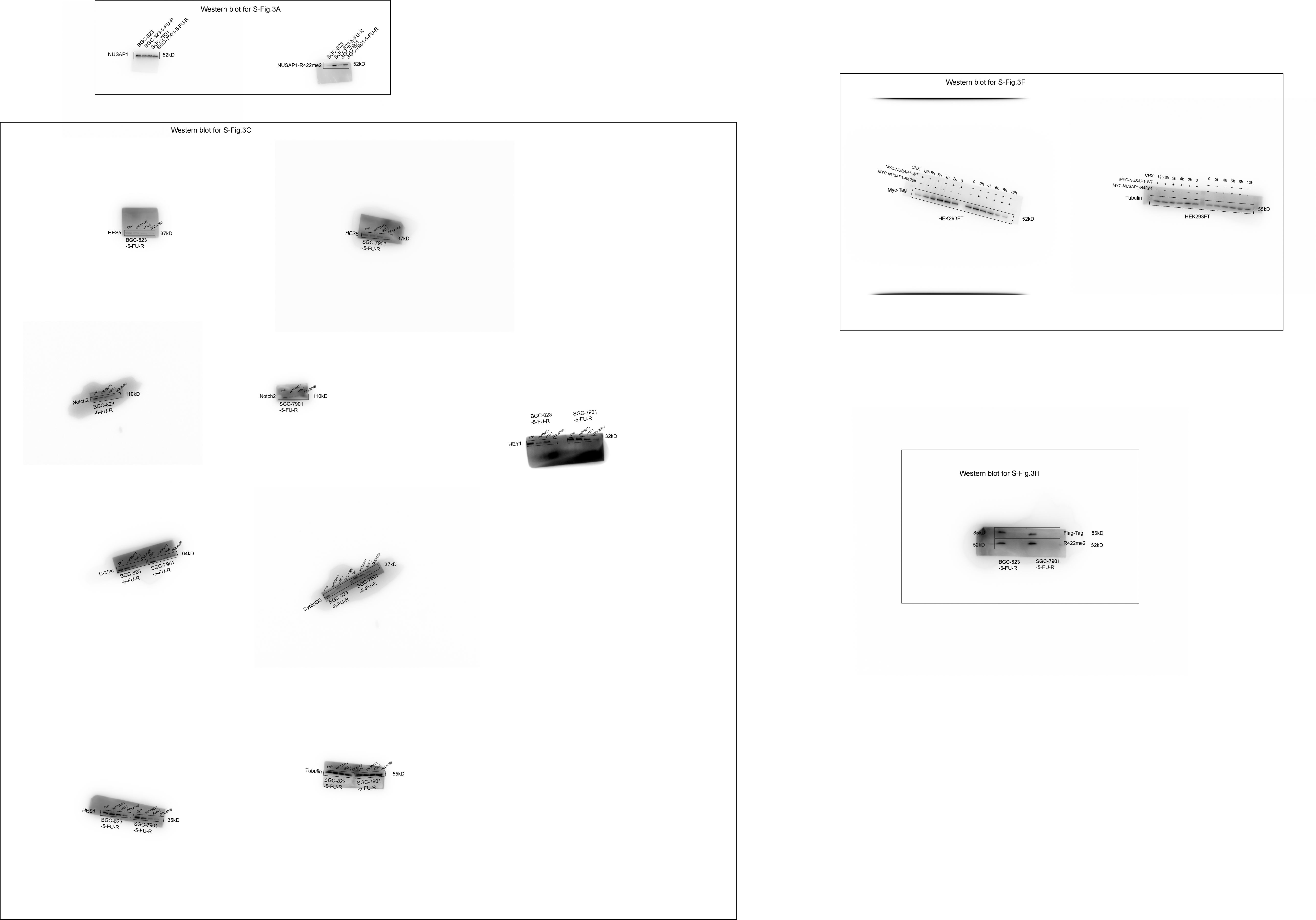

Supplement: Supplementary file 6 — Original western blot for FigureS1-S3 [file 41419_2025_7723_MOESM6_ESM.tif]
